# Salinity change evokes stress and immune responses in Atlantic salmon with microalgae showing limited potential for dietary mitigation

**DOI:** 10.3389/fphys.2024.1338858

**Published:** 2024-02-12

**Authors:** Doret R. van Muilekom, Jonas Mueller, Jacqueline Lindemeyer, Thekla Schultheiß, Edmund Maser, Henrike Seibel, Alexander Rebl, Carsten Schulz, Tom Goldammer

**Affiliations:** ^1^ Fish Genetics Unit, Institute of Genome Biology, Research Institute for Farm Animal Biology (FBN), Dummerstorf, Germany; ^2^ Department for Marine Aquaculture, Institute of Animal Breeding and Husbandry, Kiel University, Kiel, Germany; ^3^ Fraunhofer Research Institution for Individualized and Cell-Based Medical Engineering IMTE, Aquaculture and Aquatic Resources, Büsum, Germany; ^4^ Institute of Toxicology and Pharmacology for Natural Scientists, University Medical School Schleswig-Holstein, Kiel, Germany; ^5^ Faculty of Agriculture and Environmental Sciences, University of Rostock, Rostock, Germany

**Keywords:** microalgae, salmon, smoltification, salinity, immunity, functional feed

## Abstract

Smoltification was found to impact both immune and stress responses of farmed Atlantic salmon (*Salmo salar*), but little is known about how salinity change affects salmon months after completed smoltification. Here, we examined (1) the effect of salinity change from brackish water to seawater on the stress and immune responses in Atlantic salmon and (2) evaluated if functional diets enriched with microalgae can mitigate stress- and immune-related changes. Groups of Atlantic salmon were fed for 8 weeks with different microalgae-enriched diets in brackish water and were then transferred into seawater. Samples of the head kidney, gill, liver and plasma were taken before seawater transfer (SWT), 20 h after SWT, and 2 weeks after SWT for gene-expression analysis, plasma biochemistry and protein quantification. The salmon showed full osmoregulatory ability upon transfer to seawater reflected by high *nkaα1b* levels in the gill and tight plasma ion regulation. In the gill, one-third of 44 investigated genes were reduced at either 20 h or 2 weeks in seawater, including genes involved in cytokine signaling (*il1b*) and antiviral defense (*isg15, rsad2, ifit5*). In contrast, an acute response after 20 h in SW was apparent in the head kidney reflected by increased plasma stress indicators and induced expression of genes involved in acute-phase response (*drtp1*), antimicrobial defense (*camp*) and stress response (*hspa5*). However, after 2 weeks in seawater, the expression of antiviral genes (*isg15, rsad2, znfx1*) was reduced in the head kidney. Few genes (*camp, clra, c1ql2*) in the gill were downregulated by a diet with 8% inclusion of *Athrospira platensis*. The results of the present study indicate that salinity change months after smoltification evokes molecular stress- and immune responses in Atlantic salmon. However, microalgae-enriched functional diets seem to have only limited potential to mitigate the related changes.

## 1 Introduction

Atlantic salmon (*Salmo salar)* is an anadromous salmonid of high economic value (Forseth et al., 2017). Global salmon production of approximately ∼2.6 million tons has stagnated over the last years (Glover et al., 2020) as high mortalities and increasing sea lice infestations of salmon as well as negative environmental impacts caused by nutrient pollution and escapes have hampered further growth of the industry (McGinnity et al., 2003; Quiñones et al., 2019). Traditionally a large part of the production cycle of Atlantic salmon takes place in sea cages, while more recently many producers turn towards using land-based recirculating aquaculture systems (RAS; Dalsgaard et al., 2013). Before Atlantic salmon can be transferred into seawater they have to undergo different physiological and morphological changes known as smoltification to prepare for life in seawater (Hoar, 1988; McCormick et al., 1998). Smoltification not only affects the physiology, but also the immune system of salmon, which manifests in decreased levels of total serum protein and immunoglobulin M (IgM) (Melingen et al., 1995) as well as reduced expression of multiple immune genes in smolts (Johansson et al., 2016). The persistence of these effects after transfer to seawater, coinciding with an increased abundance of pathogens in the marine environment, likely explain some of the high disease-related mortalities seen during the first weeks in seawater (Soares et al., 2011; Bang Jensen et al., 2020). Nowadays, salmon are often reared as larger smolts or post-smolts in RAS on land to shorten the overall time they spend in the sea. As the RAS environment can be fully controlled, many environmental factors such as salinity and water velocity can be adapted for optimal growth and welfare (Ytrestøyl et al., 2020; Mortensen et al., 2022). After their smoltification in freshwater, an acclimation period in brackish water could alleviate the salmon’s transition to salt water and improve their overall health and performance (Ytrestøyl et al., 2020; Ytrestøyl et al., 2022).

Besides changes in the rearing environment, the use of functional feeds can improve the fish’s ability to cope with environmental stressors, stimulate immunity and eventually improve production performance (Dawood et al., 2018; Marimuthu et al., 2022). Microalgae, which have known immune-stimulatory properties in different fish species (Morris et al., 2007; Abdel-Tawwab and Ahmad, 2009; Cerezuela et al., 2012; Zhang et al., 2014; An et al., 2016; Luo et al., 2018), are promising candidates for functional feeds for Atlantic salmon. For example, the addition of *Chlorella* sp. to diets increased the levels of IgM and IgD antibodies in blood, and the cytokine levels of interleukin-22 (Il-22) and C-C motif chemokine ligand 5 (Ccl-5) in the kidney and liver of Gibel carp *Carassius gibelio* (Zhang et al., 2014). Supplementation with the microalgae *Phaeodactylum tricornutum* increased phagocytic activity and *Nannochloropsis gaditana* enhanced the expression of β-defensin in the head kidney of gilthead seabream *Sparus aurata* (Cerezuela et al., 2012). Furthermore, it has been shown that diets containing *Tetraselmis* sp. lowered cortisol levels after confinement stress in gilthead seabream (Pereira et al., 2020) and diets containing *Arthrospira platensis* counteracted the increasing glucose concentrations after air exposure and enhanced antioxidant activity in gilthead seabream (de Mattos et al., 2019).

A variety of phenotypic and molecular markers are used to assess the health and welfare of farmed fish (Barreto et al., 2022). Gene expression profiling allows the detection of both systemic and local immune responses in key immune organs. Recent studies on Atlantic salmon have revealed a variety of genes that can be used to assess the salmon’s immune status (Krasnov et al., 2020; Lund et al., 2022). Accordingly, expression analysis of stress- and immune-related genes can complement traditional indicators such as growth performance, organ health and plasma indicators. In this study, three key immune organs with varying functions in stress and immune response were analyzed. The head kidney is a major lymphoid and myeloid organ that controls the systemic stress and immune response of fish (Press et al., 1994; Press and Evensen, 1999). The gill is part of the mucosa-associated lymphoid tissue and plays a crucial role in the local immune response, as it is in direct contact with the environment (Haugarvoll et al., 2008; Rességuier et al., 2020). Lastly, the liver was included as the main metabolizing organ to gain insight into the fish’s oxidative status (Birnie-Gauvin et al., 2017; Hussein et al., 2023).

Given the lack of knowledge on how salinity change affects stress physiology and immunity of Atlantic salmon and its potential dietary mitigation, the aim of our study was twofold: (1) assessing the effect of salinity change to seawater following acclimation of Atlantic salmon to brackish water on the stress and immune response and (2) evaluating whether microalgae enriched functional diets can mitigate stress and immune-related changes following salinity change.

## 2 Material and methods

### 2.1 Experiment and sampling

The experimental setup has been described in detail in Mueller et al. (2023). Briefly, two identical RAS (7.6 m^3^, turnover rate 4 times h^−1^) at the facilities of the Fraunhofer IMTE, Büsum, Germany were used for the trial, where one was operated on brackish water (BW; 13.0 ± 0.8 psu, 13.5 ± 0.4°C, 7.3 ± 0.1 pH, 10.3 ± 0.2 mg/L O_2_, 0.12 ± 0.07 mg N/L total ammonia 0.06 ± 0.01 mg N/L NO_2_^−^) while the other was operated on full marine conditions (SW; 31.8 ± 0.5 psu, 13.4 ± 0.3°C, 7.2 ± 0.1 pH, 10.4 ± 0.3 mg/L O_2_, 0.12 ± 0.07  mg N/L total ammonia 0.07 ± 0.02 mg N/L NO_2_^−^). Atlantic salmon smolts, raised in freshwater, were obtained from Jurassic Salmon, Karnice, Poland and acclimated in brackish water for 2 months in the RAS prior to the start of the experiment. Following acclimation, the fish were fed six different functional diets for 8 weeks in brackish water until 2 weeks after transfer to seawater. This time-interval was chosen as one of the most critical phases during the production of Atlantic salmon (Bang Jensen et al., 2020; Sommerset et al., 2020). The fish were hand-fed twice daily (8 a.m. and 2 p.m.) until apparent satiation. The functional diets were designed to be isonitrogenous and isoenergetic (dry matter basis) and were enriched with one of the following microalgae: *Chlorella vulgaris* (either intact cell wall, CVI or broken cell wall, CVB), *Tetraselmis chuii* (TC), *Athrospira platensis* (AP) or *Schizochytrium limacinum* (SL) at an inclusion level of 8%. A diet without added microalgae served as a control (CD; Table 1). The microalgae were chosen based on their production volume, allowing for industrial upscaling, as well as containing a diversity of functional compounds. The inclusion level of 8% reflected a compromise of formulating diets without making major adjustments to a variety of ingredients within the feed formulation, as well as making sure sufficient amounts of functional components are available for the fish. At the start of the experiment 28 fish were stocked into each tank (with three tanks assigned to each treatment), but due to serial samplings, 19 fish per tank were transferred to seawater.

**TABLE 1:** Formulation of experimental diets in g/100 g dry matter (DM) as well as crude composition in % DM.

Ingredients (g/100 g DM)	CD	CVI	CVB	TC	AP	SL
Fish meal^a^	15.0	15.0	15.0	15.0	15.0	15.0
Microalgae	0.0	8.0	8.0	8.0	8.0	8.0
Blood meal^b^	6.0	6.0	6.0	6.0	6.0	6.0
Gelatine^c^	5.0	5.0	5.0	5.0	5.0	5.0
Pea protein isolate^d^	14.0	14.0	14.0	14.0	14.0	14.0
Soy protein concentrate^e^	11.0	11.0	11.0	11.0	11.0	11.0
Wheat gluten^f^	12.0	7.2	6.5	8.4	5.3	9.4
Wheat starch^f^	21.4	18.4	18.9	17.9	20.2	19.3
Canola oil^g^	5.5	5.5	5.5	6.1	5.5	2.5
Fish oil^a^	6.0	6.0	6.0	6.0	6.0	6.0
Methionine^h^	0.1	0.1	0.1	0.1	0.1	0.1
Vitamin and mineral premix^d^	0.5	0.5	0.5	0.5	0.5	0.5
CaHPO4^i^	2.0	2.0	2.0	2.0	2.0	2.0
Bentonite^j^	1.5	1.3	1.5	0	1.5	1.2
Crude composition (% DM)						
Dry matter (%)	90.03	91.52	91.59	92.90	92.08	93.02
Protein (%)	51.04	51.26	51.00	50.93	51.11	51.38
Fat (%)	16.18	16.38	16.60	16.87	16.19	15.11
Ash (%)	7.47	8.15	8.28	8.61	8.07	7.72
Crude energy (MJ/kg)	22.72	22.71	22.68	22.72	22.69	22.70

CD, control diet, CVI *C. vulgaris* intact, CVB *C. vulgaris* broken, TC *T chuii*, AP *A. platensis*, SL *S. limacinuum.*

^a^ Bioceval GmBH & Co. KG, Cuxhaven; Germany.

^b^ Saria SE & Co. KG, Selm, Germany.

^c^ Gustav Ehlert GmbH & Co. KG, Verl, Germany.

^d^ Emsland-Aller Aqua GmbH, Golßen, Germany.

^e^ EURODUNA Rohstoffe GmbH, Barmstedt, Germany.

^f^ Kröner-Stärke GmbH, Ibbenbüren, Germany.

^g^ Cargill GmbH, Riesa, Germany.

^h^ Evonik Industries AG, Essen, Germany.

^i^ Lehmann & Voss & Co. KG, Hamburg, Germany.

^j^ Del Lago Bentonite, Castiglioni Pes y Cía., Buenos Aires, Argentina.

Samples were collected after 8 weeks of feeding the experimental diets in brackish water (T1), 20 h following transfer to seawater (T2) and 2 weeks in seawater (T3). These timepoints include both the acute stress and immune response (T2) as well as adaptive immune response and further adaptive responses (T3) to salinity change. Each time nine fish per diet (three per tank) were randomly sampled. The salmon were netted from the tanks and euthanized with MS-222 (0.3 mg/L). Length and weight were recorded and a 2 mL blood sample was withdrawn in heparinized syringes by caudal vein puncture. Blood samples were immediately centrifuged at 4000 *g* for 8 min and plasma aliquots flash-frozen at −80°C, to be used for the determination of plasma metabolites. The liver and spleen were removed, weighed and a small piece of the liver was flash-frozen on dry ice for determination of protein concentrations. The first left gill arch and the head kidney were sampled for gene-expression analysis and flash-frozen in a RNase-free tube in liquid nitrogen. Somatic indices were calculated as follows:

CF (Fulton´s condition factor) = weight/fish length^3^ *100.

HSI (hepatosomatic index) = liver weight /fish weight * 100.

SSI (spleen somatic index) = spleen weight /fish weight * 100.

### 2.2 Plasma enzymes and metabolites

Plasma glucose, Na^+^ and Cl^−^-ions in plasma samples were measured on a Fuji Dry Chem NX500i (Fujifilm, Tokyo, Japan) using kits of the manufacturer. Plasma cortisol was determined by an enzyme-linked immunosorbent assay (ELISA) Kit (Demeditec Diagnostics GmbH, Kiel, Germany) following the manufacturer’s instructions.

### 2.3 Western blots

Liver samples (pooled per tank) were processed to extract total protein based on radioimmunoprecipitation (RIPA) following the manufacturer’s instructions (RIPA Lysis Buffer System, Santa Cruz Biotechnology, Dallas, Texas, United States). Cu, Zn superoxide dismutase (Sod1) and myeloperoxidase (Mpo) proteins were analyzed using SDS-PAGE and Western blot techniques. The protein ß-actin (Actb) was used as a loading control. A no template control and a positive control per antibody were included, with *Danio rerio* liver used for Mpo, bovine liver for Sod1, and HEK-293 cells for ß-actin. To process 20 µg of total protein, reducing conditions were applied with SDS sample and reducing buffer (both TruPAGE, Sigma-Aldrich, Schnelldorf, Germany) for 10 min at 70°C. SDS-PAGE was performed with precast 4%–12% gradient gels, TruPAGE running buffer, and antioxidant (Sigma-Aldrich) in an Xcell SureLock Mini-Cell (Thermo Fisher Scientific, Waltham, Massachusetts, United States). The proteins were then electro-transferred to a PVDF membrane. The membrane was cut horizontally for parallel protein detection of Mpo and Sod1. Primary antibody incubation was carried out overnight at 4°C using Mpo antibody (ab210563, Abcam, 1:5000 dilution in PBS-T containing 2.5% skim milk) and Sod1 antibody (NBP2-24915, Novus Biologicals, 1:500 dilution in PBS-T containing 2.5% skim milk). Using a dilution of 1:5000, the secondary antibody anti-rabbit IgG conjugated HRP (sc-2357, Santa Cruz Biotechnology) was incubated at room temperature for 90 min. ECL detection reagents and chemiluminescence film (both GE Healthcare, Amersham, United Kingdom) were used for detection, with 40 s of exposure time for both Mpo and Sod1. To detect Actb, the antibodies were stripped in 100 mM glycin buffer (pH 2.5). The membrane was then incubated overnight at 4°C in a 1:5000 dilution of Actb antibody (NB600-503, Novus Biologicals, Wiesbaden, Germany). Protein abundance was quantified by densitometric analysis of the protein bands using GIMP and normalized to the concentration of the reference protein Actb.

### 2.4 RNA isolation and multiplex gene-expression profiling

TRIzol (Thermo Fisher Scientific) was used to extract total RNA, which was then purified with the ISOLATE II RNA Micro Kit (Meridian Bioscience Inc., Cincinnati, Ohio, United States). NanoDrop One (Thermo Fisher Scientific) was used to determine the concentration of the isolated RNA. High-quality RNA was then reverse-transcribed into cDNA utilizing the Reverse Transcription Master Mix (Standard BioTools, South San Francisco, California, United States). cDNA samples were preamplified with the Fluidigm PreAmp Master Mix before purification with exonuclease I (New England BioLabs, Frankfurt/Main, Germany). All procedures were conducted according to the manufacturer’s instructions.

Exon-skipping oligonucleotide primers for the smoltification markers *nkaα1a* (homologous to mammalian *atp1a1*) and *nkaα1b* were designed using the Pyrosequencing Assay Design software v.1.0.6 (Biotage, Uppsala, Sweden; Supplementary Table S1). To evaluate salmon immunocompetence, Krasnov et al. (2020) designed a set of genes involved in important immunological and stress-relevant pathways and functions, such as antigen presentation, T cell activity, oxidative stress, antiviral and antibacterial defense as well as stress-related pathways such as nf-κb pathway, acute phase response and heat shock protein (HSP) signaling pathway. In addition to 41 of those target genes and three reference genes (Supplementary Table S1), three more immune-specific genes were included (Pyrosequencing Assay Design software v.1.0.6) for *hamp*, *saa5*, and *sod1* (Supplementary Table S1). The LightCycler-96 system (Roche Diagnostics International AG, Rotkreuz, Switzerland) was utilized to assess the smoltification status using the *nkaα1a* and *nkaα1b* primers (1 μL) together with 6 μL SensiFAST SYBR No-ROX Mix (Meridian Bioscience Inc.) and 5 μL cDNA in a 12-μL-reaction volume. The transcripts were amplified according to following program: initial denaturation at 95°C for 300 s, then 40 cycles including a denaturation step at 95°C for 30 s, primer annealing at 60°C for 15 s and at last, elongation step at 72°C for 15 s followed by fluorescence measurement at 72°C for 10 s. Afterwards, gel electrophoresis and melting-curve analysis were conducted to check the quality of the amplicons. Finally, the qPCR results were obtained using the LightCycler-96 analysis software v. 1.1.0.1320 (Roche Diagnostics International AG) and normalized using the geometric mean of two reference genes ribosomal protein L4 (*rpl4*) and ribosomal protein S20 (*rps20*). Standard curves of *nkaα1a* and *nkaα1b* oligonucleotides were generated (10^8^–10^3^ copies per 5 μL; *R* ^2^ > 0.99) to calculate the individual copy numbers. Multiplex immunogene-expression profiling was conducted on 48.48 Gene Expression biochips. The pre-amplified cDNA samples and primers were transferred into the sample and assay inlets of these biochips that were first primed in the MX IFC Controller (Standard BioTools). Subsequently, the concentrations of the target transcripts were quantified using the Biomark HD (Standard BioTools) according to the manufacturer’s thermal protocol “GE Fast 48 × 48 PCR + Melt v2.pcl” (application type: gene expression; passive reference: ROX; assay: single probe). By use of Fluidigm real-time PCR analysis software v3.0.2 (Standard BioTools), the raw Cq values were gathered and used for calculation of the relative expression of the target genes based on the ΔCt method. Three reference genes *actb*, *rpl4* and *rps20* were employed as internal normalizers. Throughout the calculation, the mean Cq per gene for all samples per organ served as a calibrator. The data collected for four individuals were excluded from the dataset because they exhibited signs of sickness (for example, liver cysts and/or irregular spleen) and subsequently showed abnormal immune-gene expression.

### 2.5 Statistical analysis

All statistical analyses were performed using R (v4.1.2; R Core Team 2021; Vienna, Austria) in the environment R studio. The relative gene expression values obtained from the multigene expression assays as well as the actual copy numbers from the qPCR evaluation of the smoltification markers (*nkaα1a/b*) were log2-transformed before statistical analysis. However, the copy numbers of the smoltification markers were displayed in a boxplot on a log10 scale to guide visual interpretation. Mixed effect models were defined for the response variable expression of gene of interest, plasma parameter or organ health index. For testing the expression of the smoltification markers, the model included isoform (*nkaα1a/b*) and timepoint as fixed factors and tank was included as a random factor. For the other gene expression data as well as plasma parameter the model included diet, timepoint and their interaction as fixed factors and the tank as a random factor. In case of testing the organ health index, as well as plasma ion concentrations among the diet groups, timepoint was not included in the model. Model residuals were inspected graphically and found to be normally distributed and heteroscedastic. Analysis of variance (ANOVA) was conducted followed by multiple contrast tests for heteroscedastic data (Hasler and Hothorn, 2008). This was done to compare the microalgae-enriched diets to the control within a given time point as well as within each diet for effects over time. Comparisons were considered significant at *p* < 0.05. In case an interaction effect of diet and timepoint was absent, subsequent contrasts were pooled over the remaining factor. For the analysis of liver protein concentrations (pooled on tank level), a model based on generalized least squares (GLS) was defined, which included only the fixed factors diet and timepoint and subsequently model evaluation followed the procedure described above.

To evaluate the overall effect of seawater transfer on gene expression data, principal component analysis (PCA) with time point as grouping was conducted on log2-transformed relative expression values for the 44 target genes investigated per tissue, as implemented in the ade4 package in R (Bougeard and Dray, 2018). The individual contribution of each gene to the overall separation of the time points in the PCA was visualized using factor maps implemented in the facto extra package in R (Kassambara and Mundt, 2020). Fold changes of each gene across the factor time, relative to the timepoint in brackish water (T2), were calculated from relative gene expression values. Subsequently fold changes were visualized in a hierarchically clustered heatmap for both organs separately using the ppheatmap package (Kolde, 2022). A range of 0.33 to 3-fold change based on the relative gene expression values was considered as basal levels. The relative expression levels of three differentially expressed genes in at least one microalgae group (*clra*, *c1ql2*, and *camp*) in the gill were visualized as log2-transformed relative expression in boxplots.

## 3 Results

### 3.1 Smoltification status and osmoregulation

**FIGURE 1:**
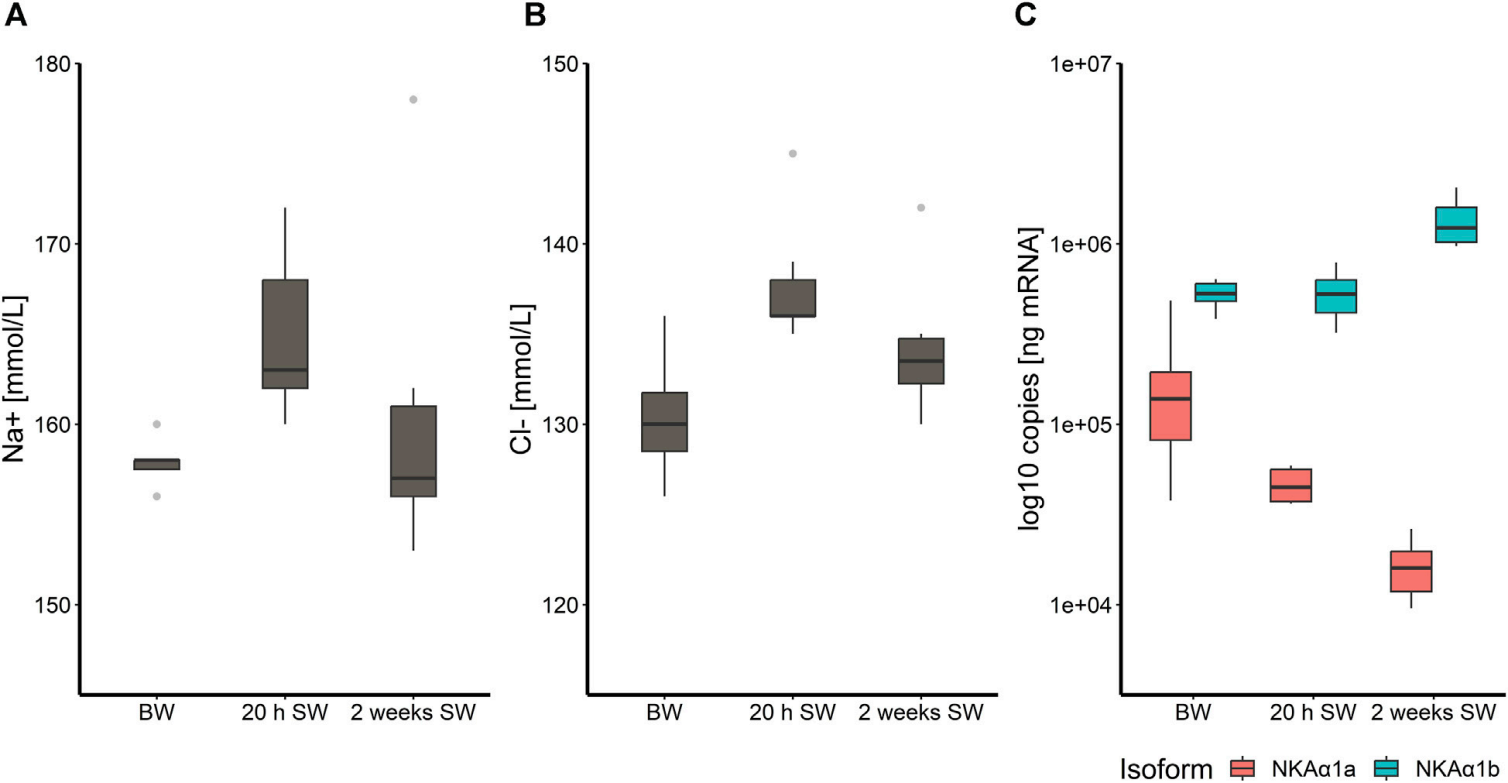
Boxplot with median and 1.5 x interquartile range whiskers showing sodium-ion **(A)** and chloride-ion **(B)** concentration in plasma of Atlantic salmon fed the control diet (CD) after 8 weeks experimental period in brackish water (T1), 20 h in seawater (T2) and 2 weeks in seawater (T3), as well as log-10- transformed copy numbers of *nkaα1a/b* transcripts for the three timepoints **(C)**; *n* = 6–8. Note that the *y*-axis in panel **(A)**, **(B)** and **(C)** does not start at zero.

Salmon acclimated to BW and transferred to full strength SW had a slight but significant increase in plasma Na^+^ and Cl^−^ concentrations (Figures 1A,B) following 20 h in SW (*p* < 0.05) in the control group. However, after 2 weeks in SW, plasma ion levels returned to levels prior to transfer. This was furthermore reflected in the expression of the two NKA isoforms in the gill. The transcripts of the “seawater isoform” *nkaα1b* were 5.1 times more abundant in the gill by the end of the brackish water phase than the “freshwater isoform” *nkaα1a* (*p* < 0.001) and this ratio increased even more 20 h and 2 weeks in SW where *nkaα1a* transcript abundance was significantly reduced further (*p* < 0.05) while *nkaα1b* transcript abundance increased (*p* < 0.001; Figure 1C).

### 3.2 Effects of salinity change on expression of immune and stress-related genes

**FIGURE 2:**
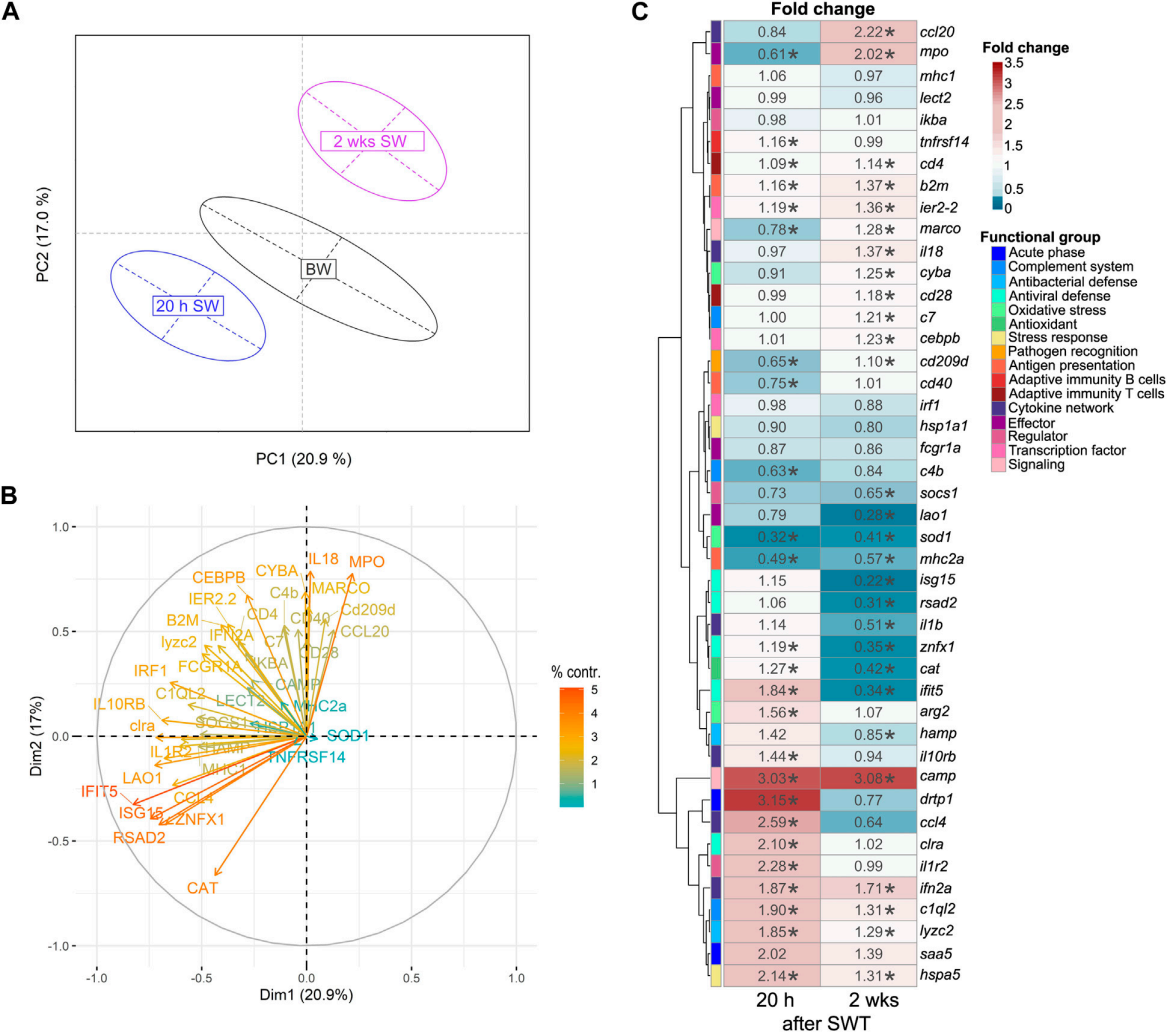
Principal component analysis **(A)** of 44 stress-and immune-regulated genes in the head kidney of Atlantic salmon after 8 weeks experimental period in brackish water (BW, T1), 20 h in seawater (T2) and 2 weeks in seawater (T3). Factor map **(B)** explaining the contribution of individual genes to the overall separation of the PCA. Length and color of the arrows indicate percent contribution of each gene. Heatmap **(C)** of fold changes for the genes investigated in relation to timepoint T1 (BW) based on relative gene expression values. Statistical significance compared to timepoint T1 was assessed using multiple contrast tests (*, *p* < 0.05). The rows represent different genes categorized into functional groups as illustrated in the legend on the right. The columns display the time points 20 h in seawater (T2) and 2 weeks in seawater (T3). Each cell is colorized based on the fold change of that gene, as visualized in the legend on the right. For each figure panel **(A–C)** *n* = 35–50 per timepoint.

**FIGURE 3:**
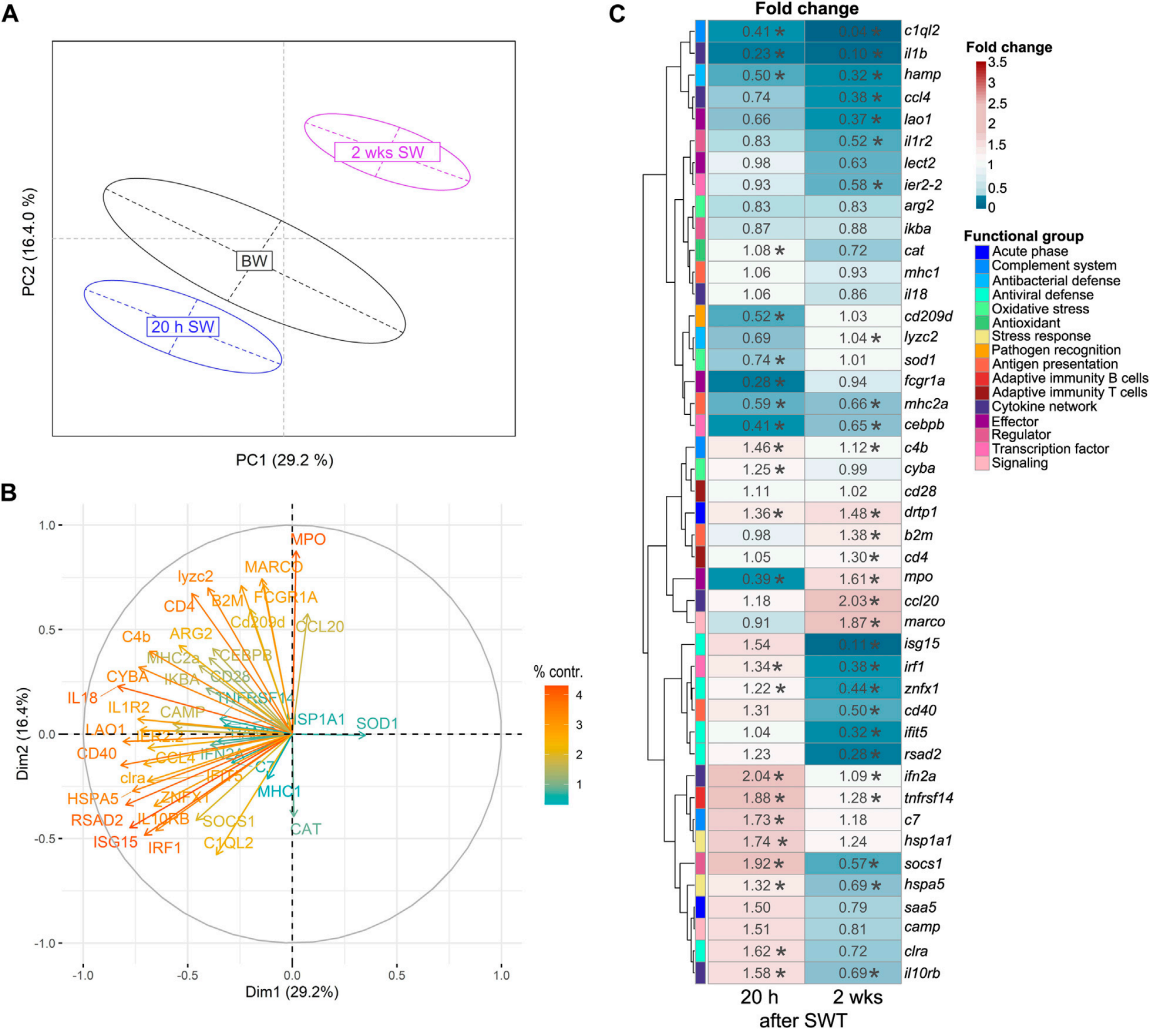
Principal component analysis **(A)** of 44 stress-and immune-regulated genes in the gill of Atlantic salmon after 8 weeks experimental period in brackish water (BW, T1), 20 h in seawater (T2) and 2 weeks in seawater (T3). Factor map **(B)** explaining the contribution of individual genes to the overall separation of the PCA. Length and color of the arrows indicate percent contribution of each gene. Heatmap **(C)** of fold changes for the genes investigated in relation to timepoint T1 (BW) based on relative gene expression values. Statistical significance compared to timepoint T1 was assessed using multiple contrast tests (*, *p* < 0.05). The rows represent different genes categorized into functional groups as illustrated in the legend on the right. The columns display the time points 20 h in seawater (T2) and 2 weeks in seawater (T3). Each cell is colorized based on the fold change of that gene, as visualized in the legend on the right. For each figure panel **(A–C)** *n* = 36–50 per timepoint.

In the head kidney, when salinity changed from BW to SW, levels of immune- and stress-related genes were significantly modulated in a time-dependent manner (Figure 2A). Principal component analysis (PCA) revealed a 21% share of variability of the first principal component for the conditions timepoint/salinity. The overall gene-expression pattern of salmon from brackish water (T1) grouped between early (T2) and the later (T3) timepoint in seawater. This pattern was driven by the differential transcript concentrations of *ifit5* (interferon-induced protein with tetratricopeptide repeats 5), *isg15* (interferon-stimulated gene 15), *rsad2* (viperin alias radical S-adenosyl methionine domain containing 2), *znfx1* (NFX1-type zinc finger containing protein), *cat* (catalase), *mpo* (myeloperoxidase) and *il18* (interleukin 18; Figure 2B). Similarly to the head kidney, the overall expression pattern in the gill was modulated in a time-dependent manner (Figure 3A). In this case, the first principal component explained 29.2% of the variance and this effect was primarily driven by *cd40* (cluster of differentiation 40)*, irf1* (interferon regulatory factor 1) and, like in the head kidney, *isg15*, *rsad2*, *mpo* and *il18* (Figure 3B).

After seawater transfer, none of the selected genes was increased in the gill above the defined threshold for basal levels (fc > 3). In contrast, the levels of several genes were significantly reduced (fc < 0.33) in the gill after SWT, such as the reduced (0.28-fold; *p* < 0.001) transcript level of *fcgr1a* (Fc gamma receptor Ia) shortly after SWT (Figure 3C). Several genes were reduced at both 20 h and 2 weeks after SWT (Figure 3C), including *c1ql2* (complement C1q-like 2; 0.41- and 0.04-fold; *p* < 0.001); *il1b* (interleukin 1β; 0.23- and 0.10-fold; *p* < 0.001); and *hamp* (hepcidin; 0.5- and 0.32-fold; *p* < 0.001). The antiviral genes *isg15, rsad2* and *ifit5* were only found significantly reduced (up to 0.11-fold; *p* < 0.001) at 2 weeks after salinity change.

The transcript profile in the head kidney differed from that in the gill. Most notably, the transcript levels of *camp* (cathelicidin) were enhanced by > 3-fold 20 h and 2 weeks after seawater transfer (*p* < 0.001, Figure 2C). Furthermore, *drtp1* (differentially regulated trout protein 1) and *saa5* (serum amyloid A-5 protein) were 2.01- to 3.15-fold induced 20 h after seawater transfer (*p* < 0.001). After salinity change, also reduced levels of genes involved in different immune pathways were observed in the head kidney. *Sod1* (superoxide dismutase 1) was reduced 20 h (0.32-fold) and 2 weeks (0.41-fold) after seawater transfer (*p* < 0.001). Furthermore, *lao1* was reduced (0.28-fold; *p* < 0.001) 2 weeks after seawater transfer. Noteworthy, the genes *isg15*, *rsad2*, *znfx1* and *ifit5* which are all involved in antiviral defense, were reduced (0.22- to 0.35-fold; *p* < 0.001) 2 weeks after seawater transfer.

### 3.3 Mitigation potential of microalgae on immune and stress responses

**FIGURE 4:**
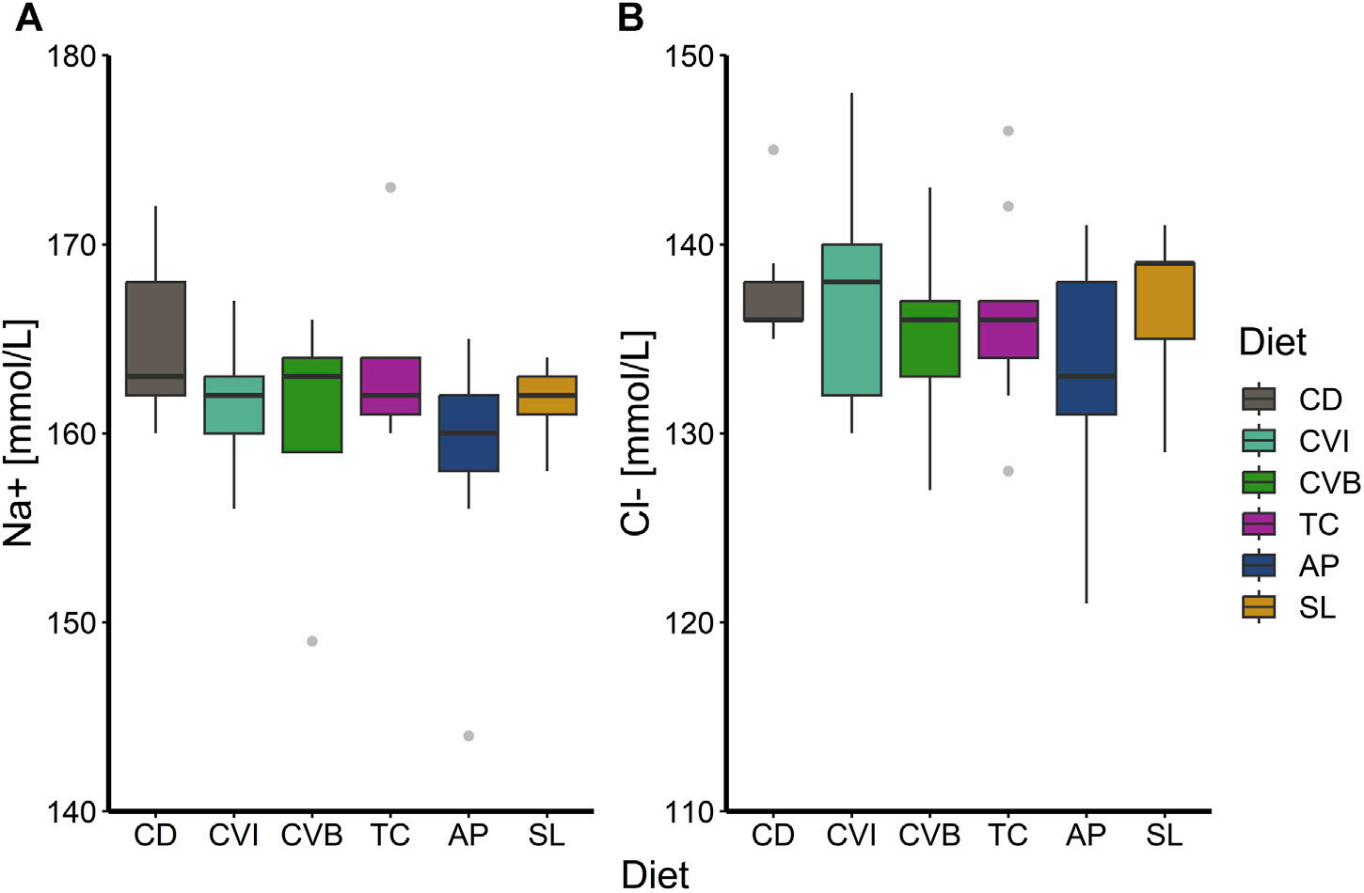
Boxplot with median and 1.5 x interquartile range whiskers showing sodium-ion **(A)** and chloride-ion **(B)** concentration in plasma of Atlantic salmon 20 h in seawater (T2) fed with the microalgae enriched diets *C. vulgaris* intact (CVI), *C. vulgaris* broken (CVB), *T. chuii* (TC), *A. platensis* (AP) or *S. limacinum* (SL) at 8% inclusion or a control diet (CD); *n* = 9. Note that the *y*-axis in panel **(A)** and **(B)** does not start at zero.

**FIGURE 5:**
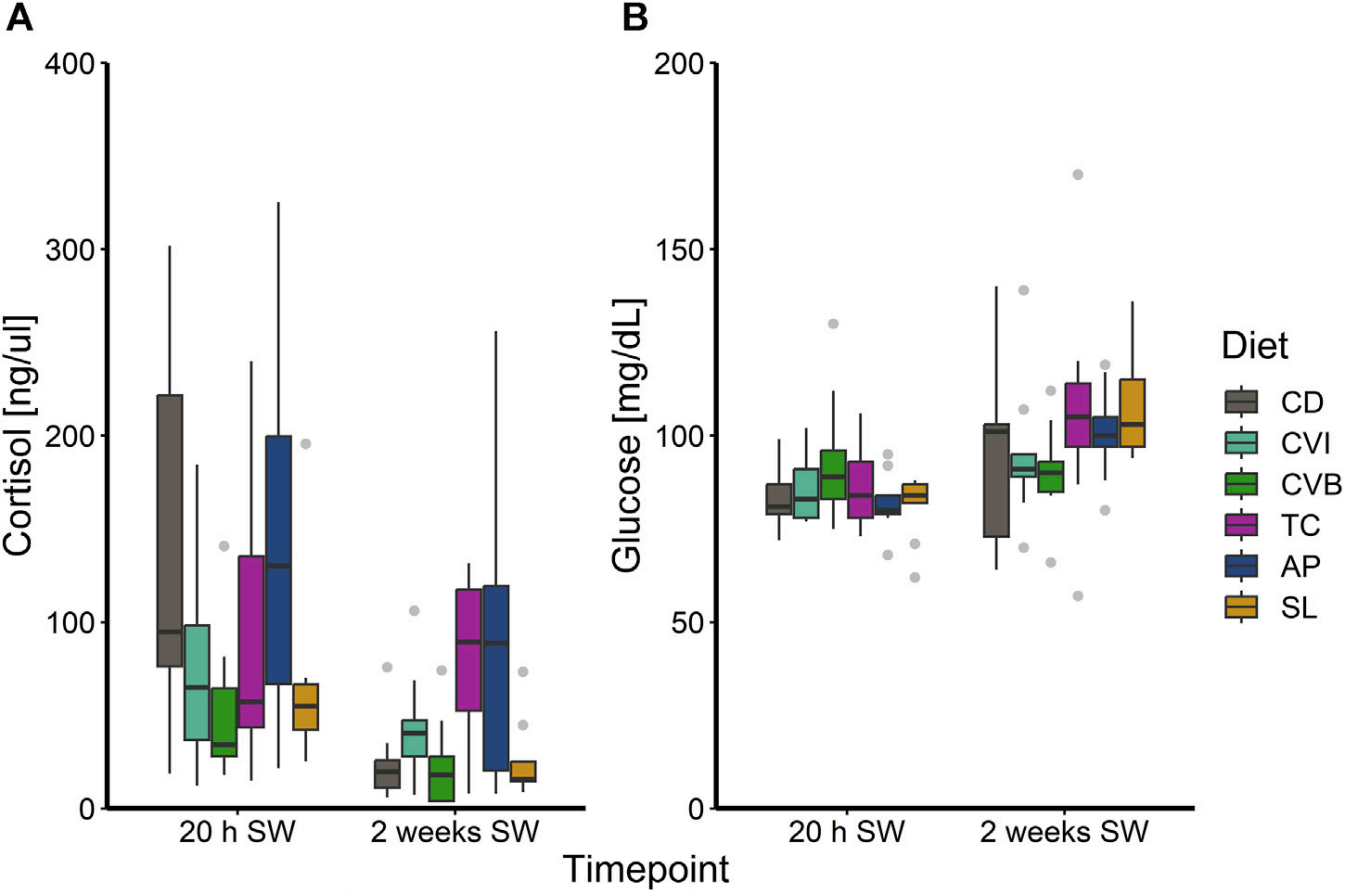
Boxplot with median and 1.5 x interquartile range whiskers showing plasma cortisol **(A)** and glucose **(B)** of Atlantic salmon 20 h in seawater (T2) and 2 weeks in seawater (T3) fed with the microalgae enriched diets *C. vulgaris* intact (CVI), *C. vulgaris* broken (CVB), *T. chuii* (TC), *A. platensis* (AP) or *S. limacinum* (SL) at 8% inclusion or a control diet (CD); *n* = 9.

**FIGURE 6:**
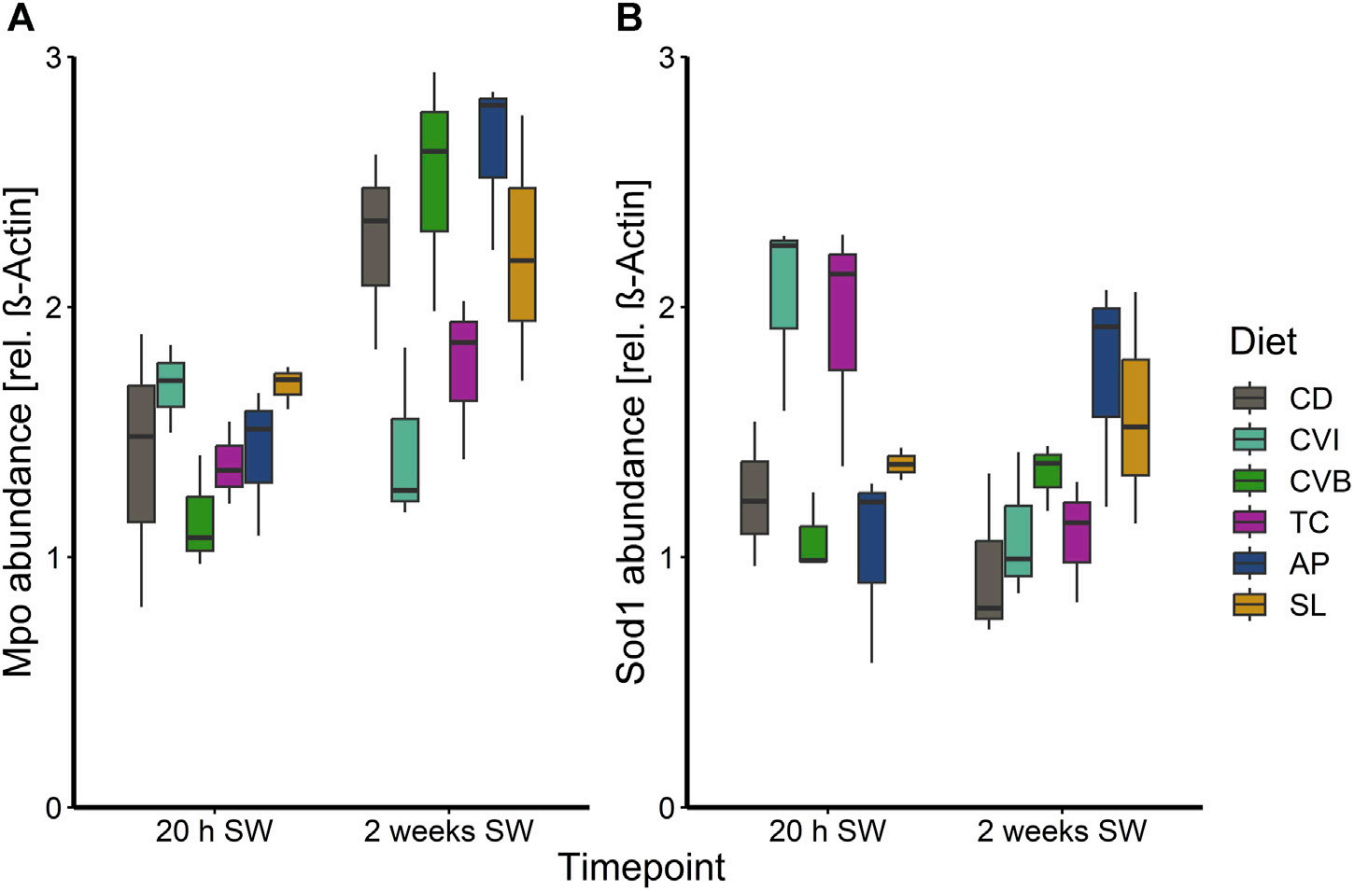
Boxplot with median and 1.5 x interquartile range whiskers showing protein abundance of myeloperoxidase **(A)** and superoxide dismutase 1 **(B)** in liver tissue of Atlantic salmon 20 h in seawater (T2) and 2 weeks in seawater (T3) fed with the microalgae enriched diets *C. vulgaris* intact (CVI), *C. vulgaris* broken (CVB), *T. chuii* (TC), *A. platensis* (AP) or *S. limacinum* (SL) at 8% inclusion or a control diet (CD); *n* = 3 pool of 3 fish per tank.

At the end of the trial in seawater, the condition of the salmon, their hepatosomatic index (HSI) and spleen somatic index (SSI) across all diet groups was similar (Table 2). Moreover, no diet-dependent effects were observed in plasma concentrations of sodium and chloride after 20 h in seawater (Figure 4). Plasma cortisol levels decreased significantly (*p* < 0.001) between timepoints in seawater for fish fed CD but remained unchanged in SW for the other diet groups (Figure 5A). Furthermore, cortisol levels after 20 h in seawater were elevated as compared to control fish in brackish water (Supplementary Figure S1). Glucose levels increased over time in seawater, which was significant for fish fed AP (*p* < 0.01) and SL (*p* < 0.001; Figure 5B). The protein levels of myeloperoxidase increased over time in seawater for fish fed CD (*p* < 0.05), CVB (*p* < 0.001) and AP (*p* < 0.01; Figure 6A). At both timepoints, the concentration of Sod1 was not significantly different in the microalgae-fed groups as compared to the control group (Figure 6B). However, for fish fed CVI and TC levels decreased over time in seawater (*p* < 0.05; Figure 6B).

**TABLE 2:** Organ indices (*n* = 9, mean ± sem) of Atlantic salmon fed with six microalgae enriched diets sampled 2 weeks in seawater (T3).

	CD	CVI	CVB	TC	AP	SL	ANOVA
CF	0.90 ± 0.01	0.86 ± 0.01	0.86 ± 0.01	0.89 ± 0.01	0.93 ± 0.01	0.87 ± 0.01	0.049
HSI (%)	1.31 ± 0.09	1.31 ± 0.1	1.51 ± 0.08	1.37 ± 0.09	1.28 ± 0.04	1.46 ± 0.07	0.635
SSI (%)	0.15 ± 0.02	0.13 ± 0.01	0.15 ± 0.01	0.13 ± 0.02	0.12 ± 0.01	0.11 ± 0.01	0.477

CD, control diet, CVI *C. vulgaris* intact, CVB *C. vulgaris* broken, TC *T chuii*, AP *A. platensis*, SL *S. limacinum*; CF (Fulton´s condition factor); HSI (hepatosomatic index); SSI (spleen somatic index).

**FIGURE 7:**
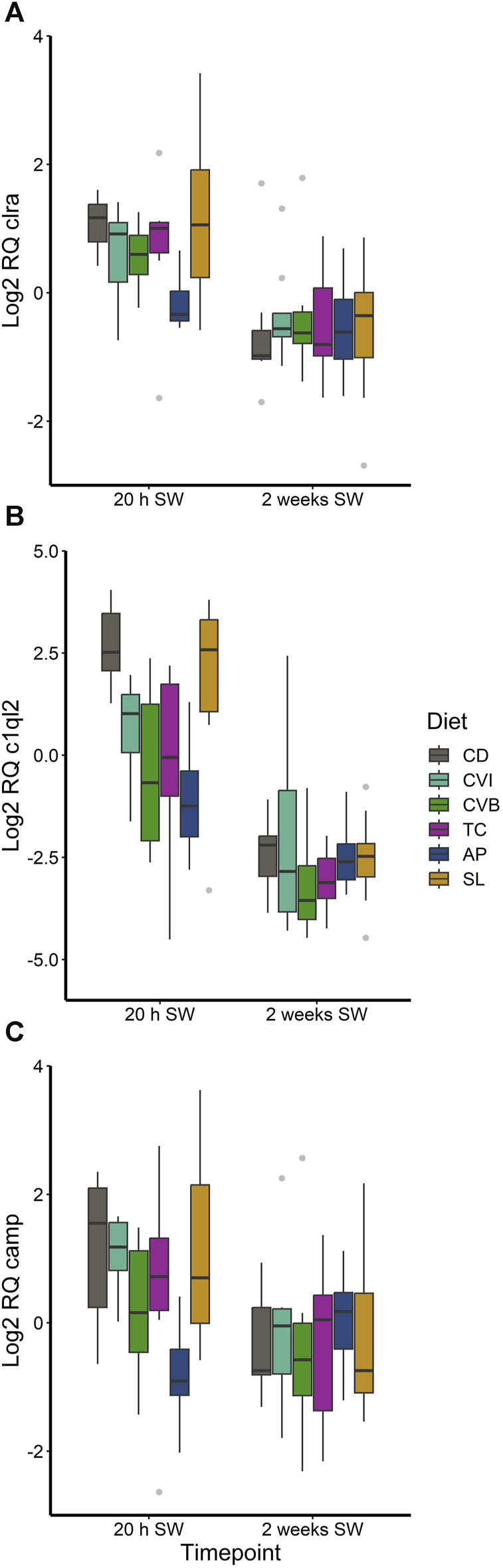
Boxplot with median and 1.5 x interquartile range whiskers showing gene expression of *clra* **(A)**, *c1ql2* **(B)** and *camp* **(C)** in the gill of Atlantic salmon 20 h in seawater (SW) and 2 weeks in seawater fed with the microalgae enriched diets *C. vulgaris* intact (CVI), *C. vulgaris* broken (CVB), *T. chuii* (TC), *A. platensis* (AP) or *S. limacinum* (SL) at 8% inclusion or a control diet (CD), *n* = 6-9.

The different diet groups followed a similar trend of gene expression over the three timepoints corresponding to brackish water phase, acute seawater transfer and 2 weeks in seawater (Supplementary Figure S2, S3). None of the investigated genes in the head kidney were differentially expressed comparing microalgae fed fish to control fish, although a diet effect in the main model was detected (Supplementary Table S2). A few genes were found to be differentially regulated in the gill of salmon fed particular microalgae as compared to the control (Supplementary Table S3; Figure 7). *Clra*, *c1ql2* and *camp* were reduced in fish fed AP 20 h after seawater transfer (Figures 7A–C). While expression of clra was reduced only modest as compared to the control (0.43-fold, *p* < 0.05), *c1ql2* (0.10-fold, *p* < 0.01) and *camp* (0.23-fold, *p* = 0.077) were more strongly reduced.

## 4 Discussion

### 4.1 Salinity change following BW acclimation does not impair the osmoregulatory ability

Upon completed smoltification salmon smolts show hypo-osmoregulatory capacity and are ready for entering seawater. However, it is generally accepted that holding salmon smolts for longer periods after smoltification in freshwater or brackish water can induce a process known as desmoltification, where the physiological ability to tolerate seawater is transiently lost (Mortensen and Damsgård, 1998; Stefansson et al., 1998). Although the salmon in the present study were reared in brackish water for several months, the fish were able to regulate their ion balance well within physiological limits upon transfer to seawater. The slight increase in plasma Na^+^ and Cl^−^ concentrations 20 h after SWT returned to basal levels after 2 weeks in SW, similar to findings from McCormick et al. (2013). In the gill, the sodium-potassium pump Na^+^/K^+^-ATPase (NKA), plays a crucial role in regulating osmolality and ion homeostasis: the NKA regulates the absorption of ions in freshwater and their secretion in seawater (Zaugg and Wagner, 1973; Evans et al., 2005). While the NKA isoform NKA α1a dominates in pre-smolts in freshwater, levels of the seawater isoform NKA α1b increase during smoltification and dominate in seawater (McCormick et al., 2009). The ability to regulate osmolality properly in seawater was also reflected by a greatly increased *nkaα1b*/*nkaα1a* ratio in the gill. In line with previous findings (Nilsen et al., 2007; McCormick et al., 2013) *nkaα1b* expression increased further in SW, while expression of *nkaα1a* decreased strongly.

### 4.2 Acute and prolonged salinity exposure induce divergent responses in the head kidney and gill of Atlantic salmon

Seawater transfer is a critical process during the life-cycle of Atlantic salmon in the wild, as well as under production. To cope with the associated altered environmental conditions, a stress response is triggered that leads to the activation of the hypothalamic-pituitary-interrenal (HPI) axis (Wendelaar Bonga, 1997). Hereby, stress hormones (such as cortisol and adrenaline) are released that induce a variety of physiological changes, allowing the fish to adapt to new the environment and return to internal homeostasis (Wendelaar Bonga, 1997). In addition to changes in, for example, oxygen uptake, metabolism or hydromineral balance, the immune system is also affected, mainly with regard to lymphocyte activities and the levels of circulating cytokines (Tort, 2011). Due to the wide range of stressors and the duration of their exposure, the stress response and also the interlinked immune response adapts accordingly, at the systemic but also at the local tissue level.

In this study, we examined how salinity change affects the expression of immune- and stress-related genes in the head kidney and gill, to get an understanding of the systemic and local immune and stress response after seawater transfer. We found that in the head kidney, the genes dominating the overall gene-expression pattern were mainly involved in antiviral defense (*ifit5*, *isg15*, *rsad2*, *znfx1*), and additionally in anti-oxidant activity (*cat*), antimicrobial defense (*mpo*) and Th1 immunity (*il18*). Transcriptional suppression of immune genes, particularly related to antiviral defense, were found during smoltification, and these effects continued after seawater transfer (Johansson et al., 2016). Interestingly, our data indicates that salinity change *per se* has an effect on antiviral immunity. This might be an evolutionary conserved response resulting from an energy-allocation trade-off upon acclimation to seawater. Concomitantly, the modulated expression of antiviral genes may be directly connected to the abundance of different viruses in freshwater and marine habitats (Maranger and Bird, 1995; Clasen et al., 2008) which requires further investigation.

Similar to the head kidney, the overall expression pattern in the gill was dominated by the antimicrobial gene *mpo* and antiviral-related genes *isg15* and *rsad2*. In addition, the antiviral gene *irf1* and the transmembrane protein-encoding gene *cd40* (alias *tnfrsf5*) majorly influenced the expression pattern of the gill. CD40 is a marker for antigen-presenting cells including neutrophilic granulocytes, dendritic cells and macrophages (Lagos et al., 2012) and could thus indicate the remodeling of the cellular immunity in the gill after salinity change, which has been found to occur in Atlantic salmon during smoltification and following seawater transfer from freshwater (West et al., 2021).

The gill is in direct contact with the environment and is therefore responsible for a local immune response. In our study, salinity change caused a suppression of several genes in the gill. Shortly after SWT, *fcgr1a* encoding an immunoglobulin Fc receptor (FcRs) was reduced. These receptors are present on the surface of phagocytes (Stafford et al., 2006). The reduction of *fcgr1a* likely indicate a decreased number of phagocytes in the gill maybe associated with an impaired antigen recognition capability. This effect is probably related to the acute stress resulting from acute salinity change, as 2 weeks later *fcgr1a* returned to the level similar to before seawater transfer. Furthermore, we observed decreased transcript levels of *c1ql2* 20 h and 2 weeks after salinity change. *C1ql2* encodes a protein which is structurally related to C1q (Köbis et al., 2017) and although its precise role is not fully defined in salmonids yet, c1ql2 might serve as a carbohydrate-binding protein (known as lectins) and bridge between innate and adaptive immunity (Köbis et al., 2017; Hillestad et al., 2020). Johansson et al. (2016) described a similar pattern in the gill of Atlantic salmon one and 3 weeks after seawater transfer, with *c1ql2* transcript numbers in gill half those relative to pre-smolts. Additionally, two genes (*il1b*, *hamp*) involved in early immune-related pathways were reduced 20 h after SWT, and still 2 weeks later. *Il1b* encodes the pro-inflammatory cytokine il-1β (Secombes et al., 2011) and *hamp* encodes for the antimicrobial peptide hepcidin (Douglas et al., 2003). Lastly, the transcript levels of the antiviral defense genes *isg15, rsad2* and *ifit5* were reduced 2 weeks after SWT and this observation may suggest that alternative defense strategies may be more beneficial in salmon exposed to salinity change. In this context it may be noted that *isg15* and *rsad2* were downregulated both in the head kidney and gill during acclimation to seawater and could thus indicate a systemic reaction.

Several genes in the head kidney were activated in our study after salinity change. Especially *camp* was greatly enhanced at both timepoints in seawater. *Camp* encodes an antimicrobial peptide with immunomodulatory activities (Scocchi et al., 2009). Furthermore, two biomarker genes (*drtp1*, *saa5*) of the acute-phase and stress response (Talbot et al., 2009; Lee et al., 2017) in fish were induced shortly after SWT. In parallel, several genes were suppressed in the head kidney after salinity change, similar as observed in the gill. *Sod1* is one of the genes, which was downregulated 20 h and 2 weeks after SWT*.* Since this biomarker gene of oxidative stress encodes a potent antioxidant, the reduction of *sod1* therefore suggests a diminished antioxidant defense or decreased amount of reactive oxygen species (ROS) in the gill after salinity change. In previous research, a change in salinity has been found to induce oxidative stress in fish (Liu et al., 2007; Lushchak, 2011). Blood samples of the freshwater fish Nile tilapia *Oreochromus niloticus* had a lower superoxide dismutase and catalase activity when fish were held in higher salinity (4 or 12 psu, respectively) than in the control group for 2 weeks (Elarabany, 2017). In contrast, in liver of anadromous chum salmon *Oncorhynchus keta,* superoxide dismutase and catalase activity was significantly enhanced in fish held for 42 days in 8, 16 and 24 psu compared to fish kept in 0 psu (Li et al., 2022). Moreover, in addition to antiviral genes (*isg15*, *rsad2*, *znfx1*, *ifit5*), *lao1* was also reduced merely 2 weeks after SWT. *Lao1* encodes for L-amino acid oxidases with antibacterial and antiparasitic activity (Kitani and Nagashima, 2020). The downregulation of these genes with important roles in immune defense may be due to the low pathogen pressure in RAS and therefore, energy could be rather directed to cope with salinity change than to maintain increased immune barriers.

In this study, expression of several immune-genes (*c1ql2, fcgr1a, hamp, ifit5, il1b, isg15, rsad2*) was reduced in the gill while in the head kidney both an induction (*camp, drtp1, saa5*) and reduction (*ifit5, isg15, lao1, sod1, znfx1*) of genes was observed after salinity change. However, these alterations in transcript levels were rather modest, possibly due to the transfer within the protected RAS environment with a lower pathogen pressure than the open marine environment. Due to different experimental setups, it is difficult to compare our results with other studies investigating freshwater to seawater transfer (Johansson et al., 2016; Karlsen et al., 2018; Lund et al., 2022), but the general pattern of gene expression is similar.

### 4.3 Microalgae have limited potential for modulating the response to changing salinity

Microalgae could contribute to improve the fish’s ability to cope with salinity, as their immune-stimulatory and stress-modulatory properties have previously been demonstrated (Cerezuela et al., 2012; Zhang et al., 2014; Raji et al., 2018). Since each of the microalgae species have their own beneficial characteristics (based on various functional compounds), this study evaluated the effects of different microalgae-enriched functional diets on the stress and immune status of salmon after salinity change. The overall growth performance among the different diet groups was not different over the 10-week trial period (Mueller et al., 2023) and furthermore similar condition and organ indices among the diet groups indicate that the incorporated microalgae did not impair growth nor energy reserves of the fish.

Cortisol is the primary stress hormone in fish and rapidly elevated in response to acute stress (Wendelaar Bonga, 1997; Barton, 2002) and alongside with glucose used as the primary stress indicator in fishes (Sopinka et al., 2016). Moreover, high cortisol levels induce hypo-osmoregulatory ability during smoltification (Langhorne and Simpson, 1986). We also observed increased levels of cortisol in post-smolts after 20 h in seawater, probably resulting from a response to salinity change rather than to handling, as cortisol in similarly handled fish that had been transferred back into brackish water, remained on a basal level. After 2 weeks in seawater, the levels of cortisol decreased, most notable for the control group and likely due to adaptation to the new environment of the fish. Notably, the functional diets did not influence this primary stress response. Interestingly glucose levels showed an opposite trend and we can only speculate why glucose levels were not elevated initially but increased slightly over time in seawater. The slightly lower glucose levels at 20 h in SW might have resulted from the usage of glucose as an energy substrate.

In addition to the transcriptional profiles in the head kidney and gills, we determined the protein levels of myeloperoxidase in the liver. Myeloperoxidase is an antimicrobial enzyme characteristic for neutrophilic granulocytes (Castro et al., 2008; Aratani, 2018; Chen et al., 2019). Fish fed with the control diet, or diets enriched with *C. vulgaris* broken and *A. platensis* showed an increase of Mpo levels over time in seawater, suggesting an increase in antimicrobial activity in the liver of these fish. The hepatic abundance of Sod1 can give insights into the antioxidant activity in the liver of the fish. The high Sod1 concentration in liver of fish fed *C. vulgaris* intact and *T. chuii* acutely after salinity change (20 h in seawater) may provide a better oxidative state during this time period but protein levels potentially decreased 2 weeks in seawater because lower ROS levels were subsequently present. In line with these results, previous research on salmon fed with a pre-extruded *Tetraselmis* diet also revealed significantly induced *sod1* expression in the liver (Sørensen et al., 2021).

Overall, microalgae enriched diets only induced subtle changes in immune-gene expression. This can be likely explained by the fact that feed intake in seawater was drastically reduced (Mueller et al., 2023), which has limited the uptake of functional compounds and their immune modulatory effects. The functional diet supplemented with *A. platensis* caused a reduction of genes in the gill involved in antimicrobial defense (*camp*) and pathogen recognition (*clra*, *c1ql2*) and we can only speculate if these modest reductions in gene expressions are reflected at the protein level. However, extracts of *A. platensis* showed strong antimicrobial activity (Metekia and Ulusoy, 2023) and this could have reduced the need to produce antimicrobial peptides as well as pathogen recognition receptors*,* explaining its reduced expression.

Altogether, the transfer from brackish water to seawater had a more pronounced effect on the immune status of the salmon than the diet, which caused subtle changes in immune-gene expression. This is an important finding for the development of future stress and immune-mitigation strategies in aquaculture.

## 5 Conclusion

The current study evaluated the effect of salinity change from brackish water to seawater on Atlantic salmon. Although the fish showed full osmoregulatory ability after rearing in brackish water, salinity change caused a reduction of immune genes in the head kidney and gill even several months after smoltification. The herein-investigated microalgae had only marginal potential to modulate the observed responses. Future research should explore whether other functional feed additives, e.g., beta-glucans can stimulate the immune system of salmon during this critical period. Furthermore, different salinity adaptation strategies and their effect on the stress and immune status of Atlantic salmon need to be evaluated experimentally.

## Data Availability

The raw data supporting the conclusion of this article will be made available by the authors, without undue reservation.
