# Supplementary material for: Salinity change evokes stress and immune responses in Atlantic salmon with microalgae showing limited potential for dietary mitigation
Source: Front Physiol. 2024 Feb 12;15:1338858. doi: 10.3389/fphys.2024.1338858 (PMC10894964; doi:10.3389/fphys.2024.1338858)
Supplement: Supplementary file 1 [file Table1.docx]

Supplementary Material

Table S1. Genes and primers used in this study.

| Gene symbol | Gene | Primer sequence 5’→3’ (sense, antisense) | NCBI-nucleotide accession codes | Primer efficiency (%) | Amplicon length (bp) |
| --- | --- | --- | --- | --- | --- |
| *Acute phase* | | | | | |
| *drtp1* | Differentially regulated protein 1 | ACCTAAAGGAAGTACAATCAGATCA,  ACAACGGTTGCATTTCAGACCC | XM_014150226;  XM_014151528 | 93.7 | 126 |
| *saa5** | Serum amyloid A-5 | TTCCACGCTCGGGGCAACTAT,  AGCTGCTGAGTCCTCATGTCC | NM_001146565 | 93.4 | 129 |
| *Complement system* | | | | | |
| *c1ql2* | C1q-like adipose protein | AGGTAGCCTTCTCAGCTTCACT,  TTCCACTCACCGGCGCCTTAA | XM_014183971; XM_014208680; XM_014187765; XR_001321949 | 98.6 | 92 |
| *c4b* | Complement component C4 | TAGCCTGTACGAAGAGGCAATC,  GAAATTGACATTCTCTCCTGGATT | XM_014162647; XM_014214474 | 83.2 | 187 |
| *c7* | Complement component C7 | CAGGTTCGCACACGGACTGTT, GTCCTGAGGAACAGCGGAACC | XM_014134762 | 98.4 | 148 |
| *hamp** | Hepcidin | GGTGCGAACGGAGGAGGTTG,  ACAGGGAGAGGTGGATCTGAC | XM_014170058; XM_014170044; NM_001140849 | 92.7 | 122 |
| *Antibacterial defense* | | | | | |
| *lyz2* | Lysozyme C II precursor | CTGCAGCCAGCTGCTAACTGA, AAGGCGCCAAGCCACCCATG | XR_001321030; BT057448; BT047934; XM_014145497 | 96.3 | 103 |
| *Antiviral defense* | | | | | |
| *clra* | C type lectin receptor A | ATCTCCAGCAGAGGAAACAAGC,  CCTGCTAAATAAGAGCACGCTCA | BT045294; BT048122; BT046430; NM_001123579 | 98.5 | 162 |
| *ifit5* | Interferon-induced protein with tetratricopeptide repeats 5-2 | ACAAGATGAGCCCTTCGGTCAA, GCCTTGTGGAGATACTCAAGAG | XM_014179290; BT046021; XM_014179291; XM_014158291 | 98.2 | 241 |
| *isg15* | Interferon-stimulated gene 15 | CCTCAACAATATCTACTGAACATATA, CACAGTGGTGTGTGGCTGAACT | NM_001123640; BT049918 | 101.6 | 101 |
| *rsad2* | Radical S-adenosyl methionine domain-containing protein 2; Viperin (rsad) | AGGTGATTTCTTGGGGAAATTAGT, TATTCGCCATACGTTTGGAACCA | BT047340; XM_014186947; NM_001140939 | 100.9 | 120 |
| *znfx1* | Zinc Finger NFX1-Type Containing 1 | AGAGAAGGGCTTTGTCCAGATAA, TACCTCTGGAAGGGCAGATCCT | XM_014168158 | 98.3 | 174 |
| *Oxidative stress* | | | | | |
| *arg2* | Arginase-2 | GACAGAAAAGGAGAGGGGTGGA,  GAAGCTCAGGTCTCCAAAATCAT | XM_014211724; BT058927 | 97.2 | 113 |
| *cyba* | Cytochrome b-245 light chain | TCATTCTCCTTACTGGTGGCATA, CTTGCCCCTTGGGTACTCTAG | XM_014175738; XM_014127044 | 100.8 | 119 |
| *sod1** | Superoxide dismutase 1 | ACCATGGTGATCCATGAGAAGG,  TAGGGCTCCCAGTAAGGGTGT | XM_014198383 | 90.3 | 150 |
| *Antioxidant* | | | | | |
| *cat* | Catalase | TTCTACACTGACGAGGGCAACT, TGGATGAAGGATGGGAACAGCA | BT059457; NM_001140302; XM_014123794; BT049990; XM_014123795; XM_014123797; XM_014173127 | 101.3 | 92 |
| *Stress response* | | | | | |
| *hsp1a1* | Heat shock 70 kDa protein | TAAACTGCAACTAACTCCAAACCA,  TGTCTGTGAAGGCCACATAGCT | XM_014192866; XM_014191271; BT059340; BT046112; XM_014203983; XM_014187508 | 95.1 | 174 |
| *hspa5* | 78 kDa glucose-regulated protein (grp78) | AGTTTGAGGAGCTCAACATGGAC,  TCCTTCACCAGCTGCTGGACC | NM_001141642, XM_014136127 | 103.2 | 154 |
| *Pathogen recognition* | | | | | |
| *cd209d* | CCAAT enhancer binding protein beta | ATCTCTCAGATGGAAGAACAAGAC, TTTAGGGAGATGTTGAGAATGACT | BT048497; NM_001141046 | 97.3 | 94 |
| *Antigen presentation* | | | | | |
| *b2m* | beta-2 microglobulin | GACTTAATGGGGGGCGTTCCA, GGCACCTTGGCACAAAGTGTTA | BT058362; BT048032; AF180487; AF180488; AF180480; AF180485; BT056667; AF180483; BT047559; XM_014184298; NM_001245913 | 101.9 | 120 |
| *cd40* | CD40 (TNFR5) | ACCACACACTAAATGCGGACCA, GTGGGTACTGACACACAGACAAT | BT049620; NM_001141236 | 96.9 | 210 |
| *mhc1* | MHC class Ia heavy chain | AAGGCTTTGTTCCGGCCAGCA, GACGTTCCTCTTCAGCACCTTC | XM_014177344; AF504022; AF504016; EF427384; EF210363; AF504019; AF504021; DQ091798; JN561333; AF504020; AF504015; AF504024; AF504018; AF504013; AF504014; AF504017; AF508864; DQ091795; L07605 | 96.4 | 123 |
| *mhc2a* | HLA class II histantigen, DP alpha chain | ATCTGGGAGCCTGAGGTGATC, GATGGTGAGGTCCTCTGTATTTAT | XM_014133066 | 99.5 | 187 |
| *Adaptive immunity B cells* | | | | | |
| *tnfrsf14* | TNF receptor superfamily member 14 | GCCAGAAATATGGGAGGTTTTGA, CCTCAGTCCTCACCAAGTCAC | XM_014147236 | 100.1 | 117 |
| *Adaptive immunity T cells* | | | | | |
| *cd28* | CD28 | TGAAGCTCTCCAGTGCCAGGC, AGGCGATGACAGTGACAGCCA | XM_014151277 | 99.6 | 134 |
| *cd4* | CD4 | CAGTCAATGTCCACCCCTCCAA, CCTGTGTCTGACAGGGCCACA | XM_014200556 ; XM_014200553 ; XM_014200557 ; XM_014200554 ; XM_014200552 ; XR_001328802; XM_014163618 ; NM_001128603 | 94.9 | 187 |
| *Cytokine network* | | | | | |
| *ccl20* | C-C motif chemokine 20-like | GTTCAACCTCAGCTGCATACGG, GCTCTGTAGTTTGTAGCCTTTTAT | XM_014191476; BT125488; BT057039; BT047144 | 96.0 | 104 |
| *ccl4* | CC chemokine 4 | ACCACCGAACATCTCTCTAGCA, GTGAACCTTGTTTGCATCAGTGT | XM_014191808 | 99.8 | 118 |
| *ifn2a* | Interferon alpha | AAAAATGAACTACAGTGCACAGGC, AAACTGGTAAGGGCGTAGCTTC | NM_001123710; NM_001123570; XM_014204440; XM_014192596; XM_014187639; XM_014192595 | 101.1 | 137 |
| *il10rb* | Interleukin-10 receptor subunit beta-like | AGTTTATCATGGATGAACACACCA, CCAGTAGGTGATGTTAAAGGTTG | XM_014174580; XM_014163988; BT059022; BT047745; XM_014174581 | 104.9 | 155 |
| *il18* | Interleukin-18 | CGGAGGTACCACACATGAATAG, GTCACTCGTAAATGTCGCCTTC | XM_014162473; BT125392; BT057200; NM_001141408; XM_014162472; XM_014162471 | 110.3 | 135 |
| *il1b* | Interleukin-1 beta | CATCATCGCCATGGAGAGGTTA, CAACTCCAACACTATATGTTCTTC | NM_001123582; XM_014143360 | 95.8 | 124 |
| *Effector* | | | | | |
| *fcgr1a* | Ig Fc receptor I | AAATAGAGGTGGACGGTGGGTG, GAAGCTCCACCCCGTCTTTGT | XM_014162559; BT058951; BT058413; XM_014162558; BT058649; XM_014214561 | 97.1 | 144 |
| *lao1* | L-amino-acid oxidase | GGCACAAGGTGACTTTAATAGAG, AAAAGAATTTTGTGAAAGCTAGGGA | XM_014213791; AB831259; XM_014213790 | 101.8 | 130 |
| *lect2* | Leukocyte cell-derived chemotaxin 2-1 | GTGGAGAGGGTCTGTGCTTTAA, TGATCCCTGGGTAGACACTTTG | XM_014211962; XM_014199364; BT059281; XM_014211966 | 98.1 | 120 |
| *mpo* | Myeloperoxidase | GAAAAGAGTCTGGAGAGAGTGC, GACTTTCTCATGGATCAGTCTCA | XM_014131319; XM_014147274; XM_014147173; XM_014128513 | 101.4 | 147 |
| *Regulator* | | | | | |
| *ikba* | NF-kappa-B inhibitor alpha | GCTAGTGGACGACAGTGGCAA, TTCTGACCACTGTAGTTTGGTGT | BT043997; BT058522; BT048185; BT048105; XM_014211117; BT125370; BT072741; XM_014193164 | 100.7 | 144 |
| *il1r2* | IL-1 receptor type II | CCAAGCAAACTGACATTGAGAAG, TGGAGTCCTTATACCACTCTATTT | NM_001145420 | 98.6 | 129 |
| *socs1* | Suppressor of cytokine signaling 1 | CACAGTCGACTGAAGGACGTC, TCACTTCCTGCCAGGCTGAAC | XM_014187410; XM_014190655 | 96.0 | 155 |
| *Transcription factor* | | | | | |
| *cebpb* | CCAAT enhancer binding protein beta | ACGACAGGGATTGCTTTGCTTTC, CCTCTCTGCTGAGTTTCGTTATG | BT049364; BT058424; XM_014146703; EU668996; NM_001139913 | 91.4 | 208 |
| *ier2-2* | Immediate early response 2-2 | ATACAGCACGACACTGATAACATA, TCAGTACAGTTTCTGGCACAATC | NM_001140121; XM_014181789 | 100.5 | 159 |
| *irf-1* | Interferon regulatory factor1 | GGGCCATGCACACAGGGAAAT, GCTTTTGTCTTTCACTTCCTCTAT | NM_001252361; XM_014136758 | 96.2 | 122 |
| *Signaling* | | | | | |
| *camp* | Cathelicidin | GGGGCAGCAAGGCCAACCAT, CCGGTCAGTGTTGAGGGTGTT | XM_014190921; NM_001123573; XM_014140492; XM_014140493 | 103.0 | 112 |
| *marco* | Macrophage receptor | TACACCTTCCAGAGCAGCGATC, CGAAAAGACTTTGTAAAGGAGGAA | XM_014164760; XM_014164759; XM_014173984; XM_014164758 | 99.8 | 129 |
| *Smoltification markers* | | | | | |
| *nka α1a** | alpha subunit of Na(+)/K(+)-ATPase-1a | CCAGGATCACTCAATGTCACTCT,  TTTTACAATGATATTAAACTCATTTGCCTTTGATAGC | XM_045722983 | 99.87 | 89 |
| *nka α1b** | alpha subunit of Na(+)/K(+)-ATPase-1b | GCTACATCTCAACCAACAACATTACAC, ATGGTGCACTCAGCTGCA | XM_014152156 | 89.84 | 91 |
| *Reference genes* | | | | | |
| *rpl4* | Ribosomal Protein L4 | CACCGCATTGAGGAGATCCCA, ATGTCGTTCCAGGCCTTGAGTT | NM_001141783.1 | 94.6 | 116 |
| *rps20* | Ribosomal Protein S20 | GCAGACCTTATCCGTGGAGCTA, TGGTGATGCGCAGAGTCTTG | PMC3031229 | 101.0 | 85 |
| *actb* | Actin Beta | CCAAAGCCAACAGGGAGAAG, AGGGACAACACTGCCTGGAT | PMC3031230 | 100.3 | 91 |

* primers designed for this study; all other primers were taken from Krasnov et al. (2020).

Table S2. Linear mixed-effect model results for 44 stress- and immune-regulated genes expressed in the head kidney of Atlantic salmon fed with six experimental diets (five microalgae enriched-diets and one control diet)

|  | ANOVA (p-value) | | |  |  |
| --- | --- | --- | --- | --- | --- |
| Gene | Diet | Timepoint | Interaction | Diet | Timepoint |
| *arg2* | 0.48 | <0.01 | 0.66 |  | T2 > T1 & T3 |
| *b2m* | 0.60 | <0.01 | 0.10 |  | T1 < T2 < T3 |
| *c1ql2* | 0.24 | <0.01 | 0.27 |  | T1 < T3 < T2 |
| *c4b* | 0.54 | <0.01 | 0.63 |  | T2 < T1 & T3 |
| *c7* | 0.24 | <0.01 | 0.19 |  | T1 & T2 < T3 |
| *camp* | 0.08 | <0.01 | 0.22 |  | T1 < T2 & T3 |
| *cat* | 0.02 | <0.01 | 0.01 |  | T3 < T1 < T2 |
| *ccl20* | 0.42 | <0.01 | 0.64 |  | T1 & T2 < T3 |
| *ccl4* | 0.02 | <0.01 | 0.98 |  | T1 & T3 < T2 |
| *cd209d* | 0.06 | <0.01 | 0.61 |  | T1 < T2 & T3 |
| *cd28* | 0.22 | <0.01 | 0.02 |  | T1 & T2 < T3 |
| *cd4* | 0.57 | 0.07 | 0.07 |  | T1 < T3 < T2 |
| *cd40* | 0.25 | <0.01 | 0.06 |  | T2 < T1 & T3 |
| *cebpb* | 0.34 | <0.01 | 0.72 |  | T1 & T2 < T3 |
| *clra* | 0.26 | <0.01 | 0.74 |  | T1 & T3 < T2 |
| *cyba* | 0.04 | <0.01 | 0.66 |  | T1 & T2 < T3 |
| *drtp1* | 0.28 | <0.01 | 0.65 |  | T1 & T3 < T2 |
| *fcgr1a* | 0.52 | 0.12 | 0.66 |  |  |
| *hamp* | 0.60 | <0.01 | 0.10 |  | T3 < T2 & T1 |
| *hsp1a1* | 0.49 | 0.42 | 0.39 |  |  |
| *hspa5* | 0.77 | <0.01 | 0.80 |  | T1 < T3 < T2 |
| *ier2-2* | 0.31 | 0.01 | 0.27 |  | T1 < T2 & T3 |
| *ifit5* | <0.01 | <0.01 | 0.12 |  | T3 < T1 < T2 |
| *ifn2a* | 0.75 | <0.01 | 0.44 |  | T1 < T2 & T3 |
| *ikba* | 0.37 | 0.89 | 0.21 |  |  |
| *il10rb* | 0.40 | <0.01 | 0.13 |  | T1 & T3 < T2 |
| *il18* | 0.90 | <0.01 | 0.29 |  | T1 & T2 < T3 |
| *il1b* | 0.32 | <0.01 | 0.50 |  | T1 & T2 < T3 |
| *il1r2* | 0.03 | <0.01 | 0.34 |  | T1 & T3 < T2 |
| *irf1* | 0.57 | 0.05 | 0.21 |  |  |
| *isg15* | 0.07 | <0.01 | 0.29 |  | T3 < T2 & T1 |
| *lao1* | 0.18 | <0.01 | 0.72 |  | T1 & T2 > T3 |
| *lect2* | 0.60 | 0.70 | 0.93 |  |  |
| *lyzc2* | 0.43 | <0.01 | 0.59 |  | T2 &T3 > T1 |
| *marco* | 0.10 | <0.01 | 0.02 |  | T3 > T1 > T2 |
| *mhc1* | 0.86 | 0.14 | 0.27 |  |  |
| *mhc2a* | 0.24 | <0.01 | 0.46 |  | T1 > T3 > T2 |
| *mpo* | <0.01 | <0.01 | 0.94 |  | T3 > T1 > T2 |
| *rsad2* | 0.17 | <0.01 | 0.70 |  | T3 < T1 & T2 |
| *saa5* | 0.43 | <0.01 | 0.03 |  |  |
| *socs1* | 0.84 | 0.01 | 0.76 |  | T3 < T1 |
| *sod1* | 0.02 | <0.01 | 0.05 |  | T1 > T3 > T2 |
| *tnfrsf14* | 0.16 | 0.02 | 0.62 |  | T1 < T2 |
| *znfx1* | <0.01 | <0.01 | 0.35 |  | T2 > T1 > T3 |

Overall model results of the linear mixed effect models with diet and timepoint as fixed factors and tank as a random factor are given in the first three columns. In the last two additional columns we display the direction of the effect, based on the subsequent multiple contrast test performed. Note that although there was an overall diet effect stated, we did not specify any effect as there was no significant difference in the microalgae fed groups as compared to the control; n = 6 - 9 per diet and timepoint.

Table S3. Linear mixed-effect model results for 44 stress- and immune-regulated genes expressed in the gill of Atlantic salmon fed with six experimental diets (five microalgae-enriched diets and one control diet)

|  | ANOVA (p-value) | | |  |  |
| --- | --- | --- | --- | --- | --- |
| Gene | Diet | Timepoint | Interaction | Diet | Timepoint |
| *arg2* | 0.12 | 0.04 | 0.85 |  |  |
| *b2m* | 0.04 | <0.01 | 0.08 |  | T3 < T1 & T2 |
| *c1ql2* | <0.01 | <0.01 | <0.01 | CD - AP overall | T1 > T2 > T3 |
| *c4b* | 0.91 | <0.01 | 0.91 |  | T2 > T3 > T1 |
| *c7* | 0.84 | <0.01 | 0.28 |  | T2 > T3 & T1 |
| *camp* | 0.13 | 0.05 | 0.08 |  | T2 > T3 |
| *cat* | 0.14 | <0.01 | 0.90 |  | T2 > T3 & T1 |
| *ccl20* | 0.91 | <0.01 | 0.59 |  | T3 > T1 & T2 |
| *ccl4* | 0.11 | <0.01 | 0.88 |  | T3 < T1 & T2 |
| *cd209d* | 0.07 | <0.01 | 0.16 | CD - CVI T3 | T2 < T1 & T3 |
| *cd28* | 0.23 | 0.01 | 0.03 |  |  |
| *cd4* | 0.14 | <0.01 | 0.90 |  | T3 > T1 & T2 |
| *cd40* | 0.36 | <0.01 | 0.16 |  | T3 < T1 & T2 |
| *cebpb* | 0.01 | <0.01 | 0.76 |  | T1 > T3 > T2 |
| *clra* | 0.03 | <0.01 | 0.42 | CD - AP T3 | T2 > T1 & T3 |
| *cyba* | <0.01 | <0.01 | 0.54 |  | T2 > T1 & T3 |
| *drtp1* | 0.49 | 0.01 | 0.76 |  | T1 < T2 & T3 |
| *fcgr1a* | 0.50 | <0.01 | 0.12 |  | T2 < T1 & T3 |
| *hamp* | 0.99 | <0.01 | 0.48 |  | T1 > T2 & T3 |
| *hsp1a1* | 0.04 | <0.01 | 0.07 |  | T2 > T1 & T3 |
| *hspa5* | 0.10 | <0.01 | 0.82 |  | T2 > T1 > T3 |
| *ier2-2* | 0.70 | <0.01 | 0.82 |  | T3 < T1 & T2 |
| *ifit5* | 0.19 | <0.01 | 0.22 |  | T3 < T1 & T2 |
| *ifn2a* | 0.04 | <0.01 | 0.25 |  | T2 > T3 > T1 |
| *ikba* | 0.81 | 0.45 | 0.01 |  |  |
| *il10rb* | <0.01 | <0.01 | 0.10 |  | T2 > T1 >T3 |
| *il18* | 0.94 | <0.01 | 0.72 |  | T2 > T3 |
| *il1b* | 0.02 | <0.01 | 0.85 |  | T1 > T2 > T3 |
| *il1r2* | 0.43 | <0.01 | 0.67 |  | T3 < T1 & T2 |
| *irf1* | <0.01 | <0.01 | 0.58 |  | T2 > T1 > T3 |
| *isg15* | <0.01 | <0.01 | 0.28 | CD - SL overall | T3 < T1 & T2 |
| *lao1* | 0.05 | <0.01 | 0.48 |  | T3 < T2 &T1 |
| *lect2* | 0.85 | 0.02 | 0.82 |  |  |
| *lyzc2* | 0.08 | <0.01 | 0.28 |  | T3 > T1 & T2 |
| *marco* | 0.34 | <0.01 | 0.80 |  | T3 > T1 & T2 |
| *mhc1* | 0.01 | <0.01 | 0.99 |  | T2 > T3 |
| *mhc2a* | 0.04 | <0.01 | 0.09 |  | T1 > T2 & T3 |
| *mpo* | 0.01 | <0.01 | 0.87 |  | T3 > T1 > T2 |
| *rsad2* | 0.66 | <0.01 | 0.73 |  | T3 < T1 & T2 |
| *saa5* | 0.08 | 0.14 | 0.06 |  |  |
| *socs1* | <0.01 | <.01 | 0.06 |  | T2 > T1 >T3 |
| *sod1* | 0.11 | <.01 | 0.35 |  | T2 < T1 & T3 |
| *tnfrsf14* | <0.01 | <.01 | 0.03 |  | T2 > T3 > T1 |
| *znfx1* | 0.01 | <.01 | 0.26 |  | T2 > T1 > T3 |

Overall model results of the linear mixed effect models with diet and timepoint as fixed factors and tank as a random factor are given in the first three columns. In the last two additional columns we display the direction of the effect, based on the subsequent multiple contrast test performed. Note that although there was an overall diet effect stated, we did not specify any effect when there was no significant difference in the microalgae fed groups as compared to the control; n = 6 - 9 per diet and timepoint.


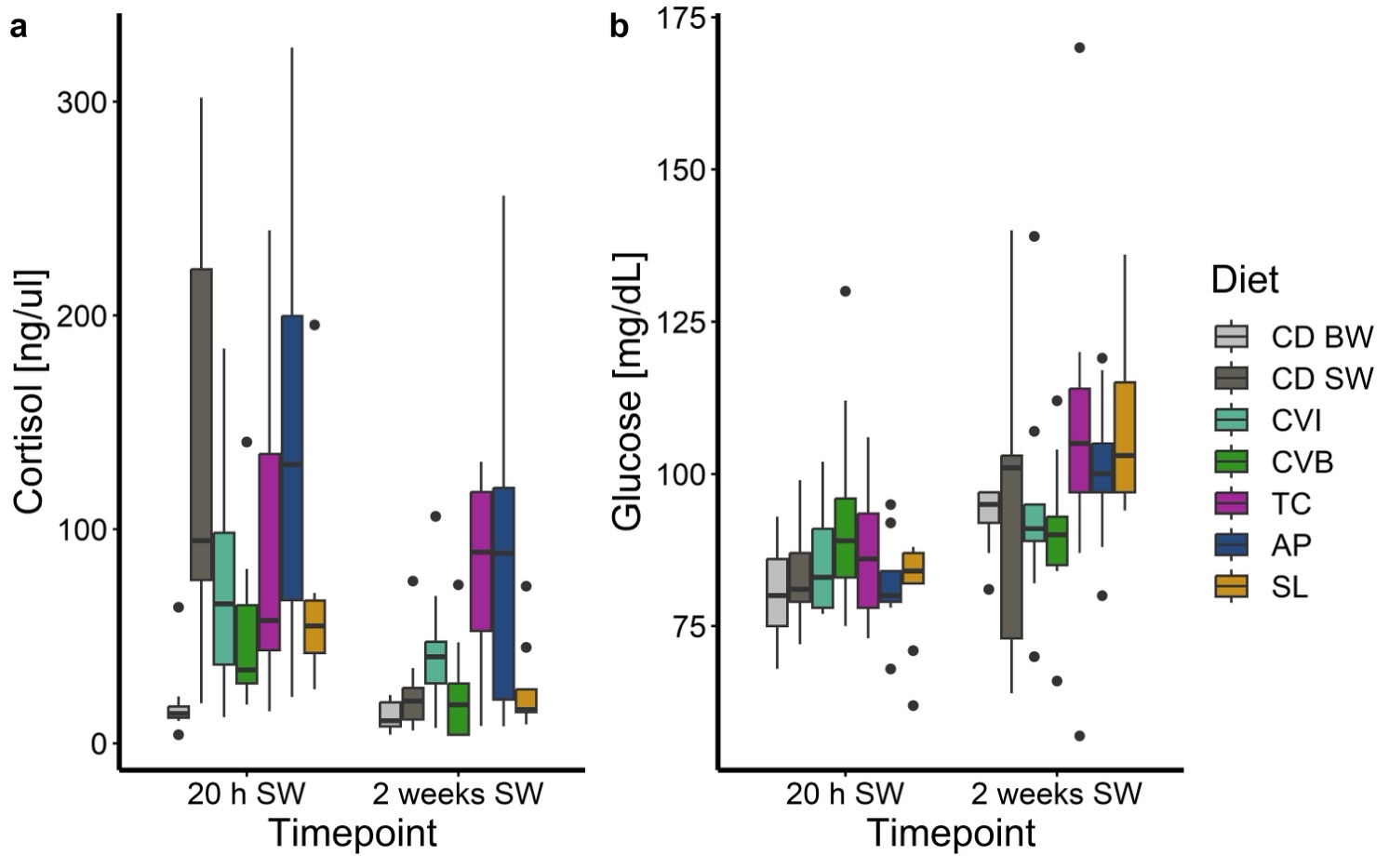


Figure S1: Boxplot with median and 1.5 x interquartile range whiskers showing plasma cortisol (a) and glucose (b) of Atlantic salmon 20 h in seawater/ brackish water (T2) and two weeks in seawater/ brackish water (T3) fed with the microalgae enriched diets *C. vulgaris* intact (CVI), *C. vulgaris* broken (CVB), *T. chuii* (TC), *A. platensis* (AP) or *S. limacinum* (SL) at 8 % inclusion or fed with control diet and transferred to brackish water (CD BW) or fed with control diet and transferred to seawater (CD SW); n = 9.

**
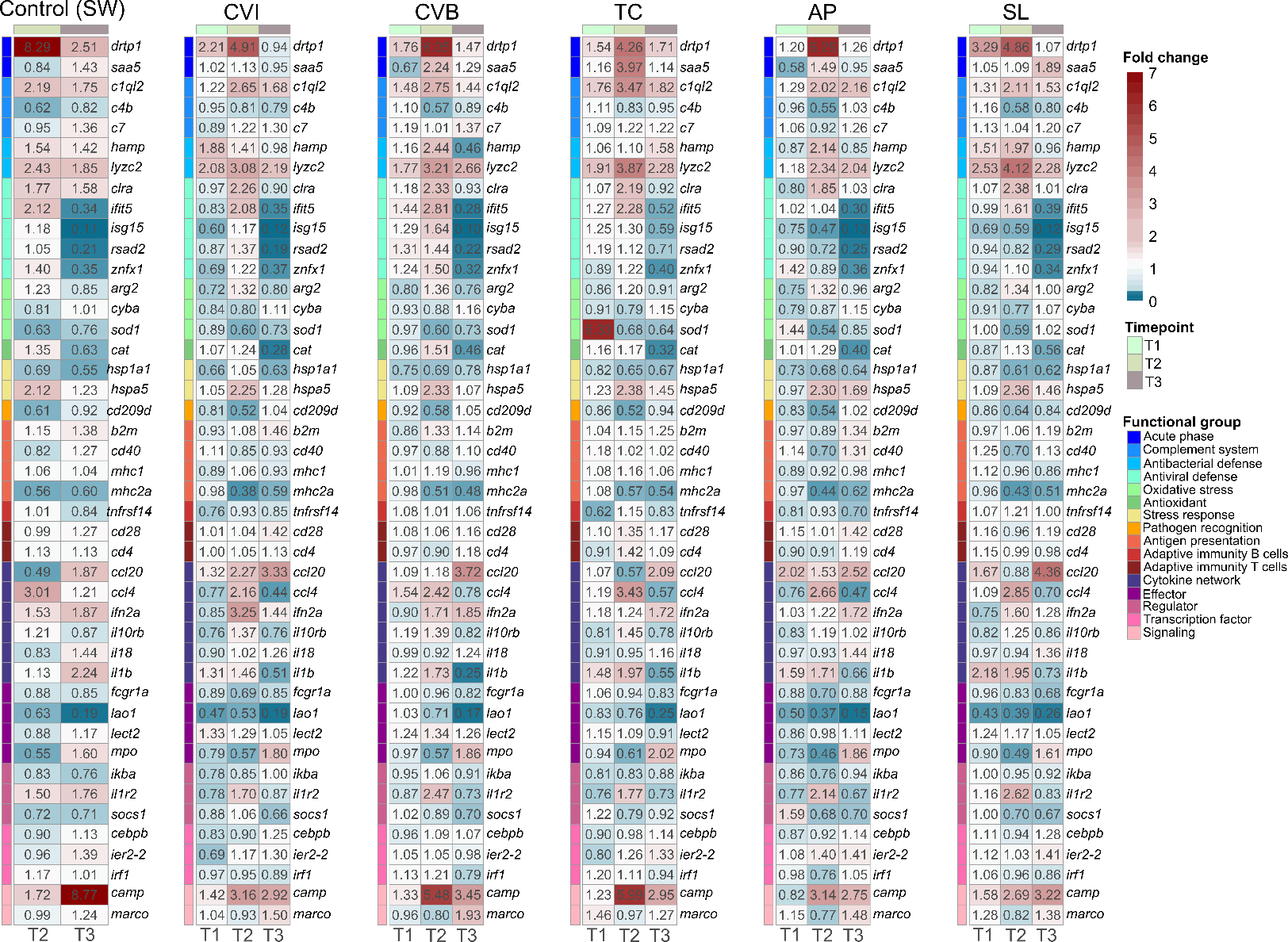
**

Figure S2: Heatmaps of fold-change values for the genes investigated in head kidney of fish fed different experimental diets supplemented with microalgae, in comparison to the expression in fish fed CD at timepoint T1. CVI = *Chlorella vulgaris* intact cell wall, CVB= *Chlorella vulgaris* broken cell wall, TC = *Tetraselmis chuii*, AP = *Athrospira platensis*, SL = *Schizochytrium limacinum*. The rows represent different genes categorized into functional groups as illustrated in the legend on the right. The columns display the time points in brackish water (T1), 20 h in seawater (T2) and two weeks in seawater (T3). Each cell is colorized based on the fold change of that gene, as visualized in the legend on the right; n = 6 - 9 per diet and timepoint.

*
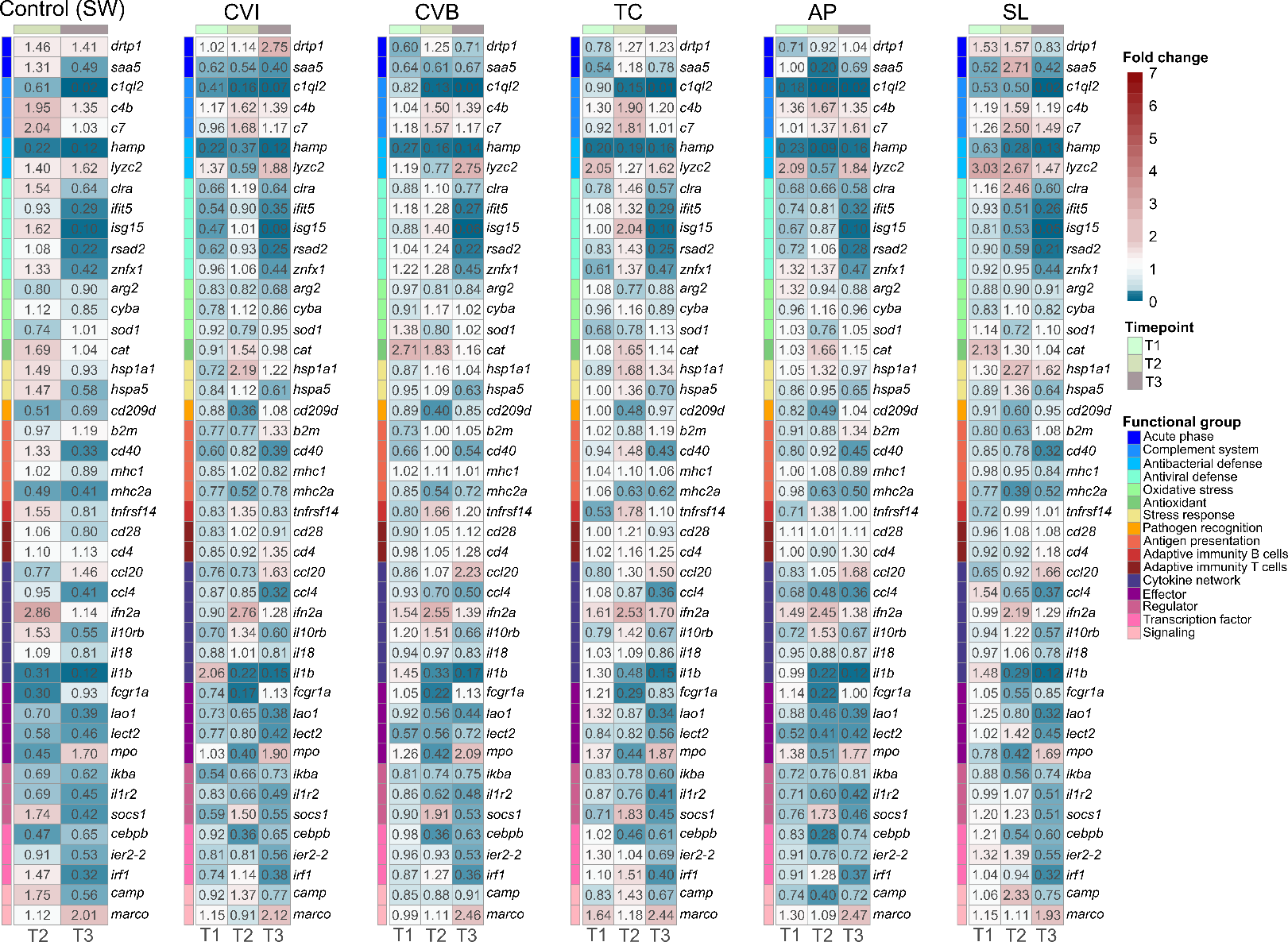
*

Figure S3: Heatmaps of fold-change values for the genes investigated in gill of fish fed different experimental diets supplemented with microalgae, in comparison to the expression in fish fed CD at timepoint T1. CVI = *Chlorella vulgaris* intact cell wall, CVB= *Chlorella vulgaris* broken cell wall, TC = *Tetraselmis* *chuii*, AP = *Athrospira platensis*, SL = *Schizochytrium limacinum*. The rows represent different genes categorized into functional groups as illustrated in the legend on the right. The columns display the time points in brackish water (T1), 20 h in seawater (T2) and two weeks in seawater (T3). Each cell is colorized based on the fold change of that gene, as visualized in the legend on the right; n = 6 - 9 per diet and timepoint.
